# Physicochemical and Antioxidant Quality Profile of Semi-Dry Tomatoes from a Local Long Shelf-Life Tomato Landrace

**DOI:** 10.3390/foods15203605

**Published:** 2026-10-10

**Authors:** Cristina Patanè, Valeria Cafaro, Daniela Romano

**Affiliations:** 1 Institute for BioEconomy (IBE), National Research Council of Italy (CNR), Via P. Gaifami 18, 95126 Catania, Italy; valeriacafaro96@gmail.com; 2 Department of Agriculture, Food, and Environment (Di3A), University of Catania, Via Santa Sofia 100, 95123 Catania, Italy; dromano@unict.it

**Keywords:** long shelf-life tomato, semi-dry tomato, lycopene, physicochemical quality, flavonoids, antioxidants

## Abstract

Long shelf-life tomato landraces, prized for the physicochemical and nutritional quality of their fruits, face the threat of genetic erosion due to their lower yields compared with commercial hybrids. Developing novel high-value products from these landraces could help preserve this valuable genetic resource. This study assessed the suitability of the local long shelf-life landrace ‘Vulcano’ for processing into a semi-dry product, in comparison with a miniplum hybrid (‘Febo’) and a Pachino-type hybrid (‘Paskualeto’), the latter renowned worldwide for its sweetness. Physicochemical traits (total solids, total soluble solids (TSS), titratable acidity (TA), reducing sugars, TSS/TA ratio (SAR)) and antioxidant properties (ascorbic acid, total phenols, flavonoids, lycopene, antioxidant activity) were evaluated in fresh fruits and in semi-dry tomatoes produced by thermo-ventilated drying, both immediately after dehydration and following seven days of cold storage. Fresh fruits of ‘Vulcano’ exhibited the highest TSS/TA ratio and lycopene content, primarily due to low titratable acidity and thick fruit skin. Dehydration significantly increased total solids, total soluble solids, reducing sugars, and phenolic compounds on a fresh-weight basis, while reducing ascorbic acid and lycopene in all cultivars. Semi-dry ‘Vulcano’ retained higher total solids and flavonoid content than the hybrids and experienced lower losses of ascorbic acid and reducing sugars during storage. Correlation and principal component analyses confirmed that dehydration, rather than cultivar, was the main factor driving quality variation. These findings support the potential of ‘Vulcano’ for producing a high-quality semi-dry product, thereby enhancing both its commercial value and the conservation of local tomato germplasm.

## 1. Introduction

The tomato is a distinctive element of the Mediterranean diet, supplying vitamins, minerals, fibre, and antioxidants—most notably lycopene—that confer considerable health benefits, including the prevention of certain types of cancer and cardiovascular diseases [1].

Among tomatoes, long shelf-life types—highly valued by consumers for their excellent taste and flavour—are distinguished by textural properties that ensure an extended post-harvest shelf-life [2]. Indeed, the thick cuticle typical of these tomatoes reduces water loss through transpiration and preserves fruit integrity [3]. These tomatoes are characteristic of Mediterranean regions, including southern Italy, and are harvested in summer, stored in well-ventilated areas, and consumed through the winter season [2]. Thanks to their high tolerance to drought, local long shelf-life tomato landraces are cultivated under rainfed conditions in semi-arid areas [3,4]. However, the low yields of these local landraces have led farmers to favour new, highly productive but often less flavoursome hybrids. Nevertheless, they represent an important source of genetic variability that must be preserved to prevent extinction and exploited, including through the development of novel products with high nutritional value for the agri-food industry.

Tomatoes are primarily consumed fresh; however, they are also processed industrially to produce a variety of products, including ketchup, tomato paste, and semi-dried tomatoes. Semi-dried tomatoes (or partially dehydrated tomatoes) are ready-to-use items that can be eaten as they are or used as a substitute for fresh tomatoes, either as starters or as ingredients in various recipes [5]. In Mediterranean countries, sun-drying is the traditional method for drying fruits and vegetables, valued for its simplicity and cost-effectiveness [6]. Sun-drying is also commonly employed to produce semi-dried tomatoes [7]. However, this method is slow and can significantly alter the fruit’s composition, while exposing the product to contamination from dust, insects, birds, and microbial spoilage [8]. Mechanical drying, widely used in industrialised countries, offers a viable alternative to sun-drying [9]. This process involves removing moisture from fruits using heated air, resulting in rapid dehydration until the desired moisture content is achieved [10]. Unlike fully dried tomatoes, semi-dried tomatoes retain a tender texture, balanced moisture levels, and high physicochemical and nutritional quality [9].

Several studies have reported the effects of partial drying on the quality of commercial tomatoes [11,12]. However, no research has addressed the impact of partial dehydration on the quality of long shelf-life tomato fruits. Nevertheless, a novel semi-dry product derived from these tomatoes could encourage a re-evaluation of low-yielding but high-quality local landraces, the loss of which may lead to genetic erosion.

The suitability of long-storage tomatoes for processing into fresh-cut products has recently been demonstrated due to the high technological, nutritional, and physicochemical quality of their fruits [13]. Therefore, this study aimed to evaluate the suitability of fruits from a local long shelf-life tomato landrace in terms of physicochemical and antioxidant quality for processing into a semi-dry product, compared with a miniplum tomato hybrid and a Pachino-type tomato hybrid—the latter well known for its high sweetness [14].

## 2. Materials and Methods

### 2.1. Plant Material

The berries of the following three tomato genotypes were examined for their physicochemical properties and antioxidant quality:

- The local landrace ‘Vulcano’, a long shelf-life tomato, originating from the homonymous Aeolian Island (Sicily, southern Italy), is part of the germplasm collection at the CNR-BioEconomy Institute in Catania, Italy. This landrace was selected for the present study due to its previously observed high physicochemical and antioxidant properties [13].
- The commercial hybrid ‘Febo’ miniplum tomato (Syngenta Seeds, Eukhuizen, The Netherlands);
- The commercial hybrid ‘Paskualeto’, a Pachino-type tomato (Syngenta Seeds, Eukhuizen, The Netherlands).

The three tomatoes were cultivated during the spring–summer season of 2021, on a flat site along the eastern coast of Sicily (southern Italy). The soil comprised 39.4% clay, 34.1% sand, and 26.5% loam, with 2.4% organic matter, a pH of 8.6, 3.2‰ N, 39 ppm P_2_O_5_, 915 ppm K_2_O, bulk density of 1.1 g cm^−3^, 27% field capacity, and an 11% wilting point.

The field trial was conducted using a randomised complete block design (RCBD) with three replicates. The tomato genotypes were randomly allocated within each replicate (block). Transplanting in the field took place in April at the four-leaf stage, with a planting density of 33,000 plants ha^−1^. Each plot measured 3.0 × 3.6 m, with a spacing of 0.75 m between rows and 0.40 m between plants within rows. Prior to transplanting, the following fertilisers were applied: 75 kg ha^−1^ of N as ammonium sulphate, 100 kg ha^−1^ of P_2_O_5_ as mineral superphosphate, and 100 kg ha^−1^ of K_2_O as potassium sulphate. One month after transplanting, a further 75 kg ha^−1^ of N as ammonium nitrate was applied as top dressing. Plants were irrigated until establishment (~80 mm in two applications), following the agronomic practices traditionally used in long shelf-life tomato cultivation [15]. An additional ~40 mm of water was applied at the time of top-dressing fertilisation. Irrigation was then suspended.

From each plot, a sample of well-ripened, disease-free tomatoes (~3 kg per plot) was harvested in mid-July for laboratory experiments. Drying and storage treatments were applied to aliquots taken from the same sample obtained from each biological replicate. An additional aliquot from the same sample was used for the fresh-product analyses.

### 2.2. Procedure for Semi-Dry Tomato Preparation

Fresh tomatoes were washed with tap water to remove any dirt, then dried with absorbent paper. They were cut longitudinally in half and divided into two batches. One batch (~300 g) was analysed immediately in the laboratory (Fresh). The second batch of cut tomatoes (~2 kg) was placed on aluminium trays and dried at 80 °C in a thermo-ventilated oven for ~5 h, until they reached ~25% of their initial water content [11]. Following the procedure described by Muratore et al. [11], each batch was weighed before drying. The initial water content (approximately 90%) was calculated from the total solids content (Section 2.3.1), and the target final weight was determined to achieve a final water content of 22.5% (i.e., equal to 25% of the initial water content). At the end of partial dehydration, some semi-dry tomatoes (~200 g) were analysed immediately in the laboratory (semi-dry T0). Other semi-dry tomatoes (~200 g) were packaged in lidded, oval-shaped PET trays under ambient atmosphere (three trays per treatment, i.e., one per open-field replicate), each with a volume (*v*) of ~ 380 mL. These were cold-stored at 4 °C in a fridge in the dark (storage RH 48%), and analysed 7 days later (semi-dry T7).

### 2.3. Laboratory Analyses

Fresh and partially dehydrated (‘semi-dry’ hereinafter) tomatoes (the latter at 0 and 7 days of storage) were homogenised for 1 min using an Ultraturrax T25 (Janke & Kunkel, Staufen, Germany) in an ice bath to prevent oxidation. A 2 g sample of each was taken to determine dry matter content by drying at 70 °C in a thermo-ventilated oven until a constant weight was achieved. Additional tomato samples (fresh and semi-dry at T0 and T7) were analysed for physicochemical and antioxidant properties. All analyses were performed in triplicate.

#### 2.3.1. Physicochemical Traits Measurements

The following physicochemical properties were measured in the laboratory: total soluble solids (TSS), titratable acidity (TA), and reducing sugars (RS).

Total soluble solids (TSS) were measured using a portable digital refractometer (Hanna Instruments Digital Brix Refractometer, HI96801, Padua, Italy) at 20 °C. The results were expressed as °Brix.

Titratable acidity (TA) was determined by titration with 0.1 M NaOH, using NaOH 0.1 M, and phenol-phthalein as the indicator. TA was expressed as g citric acid 100 g^−1^.

Reducing sugars (RS) were measured using a UV spectrophotometer (UV-30 Scan Spectrophotometer, Onda, Carpi, Italy) following the 3,5-dinitrosalicylic acid (DNS) method [16]. Briefly, a 5 g of homogenate sample was diluted with 5 mL of distilled water, vortexed, and centrifuged at 5000× *g* for 5 min. To an aliquot (100 μL) of the extract, 900 μL of distilled water and 1 mL of DNS reagent were added. The mixture was then incubated in a water bath at 90 °C for 15 min. Afterwards, the extract was rapidly cooled in a cold-water bath and mixed with 1 mL of 40% Na and K tartrate solution. Absorbance was measured at 575 nm, and the RS content was calculated using glucose (0–1 mg mL^−1^) as a standard curve (*R*^2^ = 0.99). RS content was expressed as g 100 g^−1^.

Finally, the ratio of total soluble solids (TSS) to titratable acidity (TA) (SAR, unitless) was calculated [17].

#### 2.3.2. Antioxidant Traits Measurements

The following antioxidants were measured: ascorbic acid (AscA), total phenols (TP), flavonoids (Flav), lycopene (Lyco), and antioxidant activity (AA).

Ascorbic acid (AscA) was determined by titration using 2,6-dichlorophenolindophenol as the indicator dye [18]. Ten grams of the homogenate sample were diluted with 5 mL of 3% (*w*/*v*) metaphosphoric acid and 5 mL of 8% (*v*/*v*) acetic acid in a conical flask, filtered, and then titrated with the 2,6-dichlorophenolindophenol solution. The dye had been standardised beforehand using a standard stock solution (1 mg mL^−1^) of ascorbic acid with a known concentration. The endpoint was indicated by a persistent pink colour. Results were expressed as mg of ascorbic acid per 100 g of tomato.

Total phenols (TP) were determined using the Folin–Ciocalteu colorimetric method [19]. A 2 g homogenate sample was extracted with 10 mL of 80% MeOH, incubated for 15 h at room temperature, and then centrifuged for 5 min at 5000× *g*. Subsequently, 125 μL of the supernatant were diluted with 0.5 mL of distilled water, followed by the addition of 125 μL of Folin–Ciocalteu reagent. The mixture was left to stand for a few minutes before 1.5 mL of a 7% (*w*/*v*) Na_2_CO_3_ aqueous solution was added. Distilled water was added to bring the final volume to 3 mL. Samples were incubated in the dark at room temperature for 90 min, after which the absorbance was measured at 760 nm using a spectrophotometer (UV-30 Scan Spectrophotometer, Onda, Carpi, Italy), against a standard curve of gallic acid (0–250 μg mL^−1^) (*R*^2^ = 0.99). Results were expressed as mg of gallic acid equivalent (GAE) 100 g^−1^.

Flavonoid (Flav) content was determined using the colourimetric method proposed by Ilahy et al. [20], with some modifications. Fifty microliters of an 80% methanol extract were mixed with 450 μL of distilled water, followed by the addition of 30 μL of a 5% (*w*/*v*) Na_2_CO_3_ solution. The samples were incubated at room temperature for 6 min, after which 60 μL of a 10% (*w*/*v*) AlCl_3_ solution was added. Following a second incubation at room temperature for 5 min, 200 μL of 1 M NaOH and 210 μL of distilled water were added, and the mixture was vortexed. Absorbance was measured spectrophotometrically at 510 nm against a standard curve of rutin (0–250 μg mL^−1^) (*R*^2^ = 0.99). Results were expressed as mg of rutin equivalent (RE) 100 g^−1^.

Lycopene (Lyco) content was determined using the method proposed by Anthon and Barrett [21]. Lycopene was extracted from 0.1 g of the homogenate sample in 8 mL of a solvent mixture (hexane, ethanol, and acetone in a 2:1:1 ratio, *v*/*v*/*v*). Following incubation at room temperature in the dark for 20 min, 1 mL of distilled water was added to separate the hexanic phase containing the lycopene. The mixture was then incubated again at room temperature in the dark for a further 10 min. Absorbance was measured spectrophotometrically at 503 and 444 nm [21]. Lycopene content was calculated as follows:

Lyco (mg 100 g^−1^) = [(6.95 × A_503_ − 1.59 × A_444_) × 0.55 × 537 × V/W]/10

where V (mL) is the volume of the added solvent mixture (8 mL), and W (g) is the weight of the homogenised tomato sample.

The antioxidant activity (AA, scavenging activity) was evaluated using the 2,2-Diphenyl-1-picrylhydrazyl-DPPH free radical scavenging assay [22]. A 2 g sample of tomato homogenate was mixed with 2 mL of a MeOH (80%) solution and incubated for 3 min at 4 °C. The samples were then centrifuged at 3500× *g* for 5 min, after which 50 μL of the supernatant was combined with 1 mL of freshly prepared 0.1 mM DPPH methanolic solution and incubated for 30 min at room temperature in the dark. Absorbance was measured spectrophotometrically at 517 nm, using DPPH without the tomato extract as a blank. A standard curve was generated using various concentrations of Trolox (0–600 µmol L^−1^) with an *R*^2^ value of 0.99. Antioxidant activity was expressed as μmol Trolox equivalent (TE) g^−1^.

### 2.4. Data Analysis

Initially, a one-way analysis of variance (ANOVA) was employed to assess the effects of ‘*cultivar*’ on the physicochemical and antioxidant properties of the product, prior to dehydration treatment (fresh product). Subsequently, a two-way ANOVA was performed, considering ‘*cultivar*’ (*C*), ‘*dehydration*’ (*D*), and their interaction (*C* × *D*) as sources of variation. In the statistical analysis, each field replicate was treated as an independent biological replicate (*n* = 3). For each biological replicate, analyses were conducted in triplicate in the laboratory and regarded as technical replicates; these were averaged prior to statistical analysis. ‘*Cultivar*’ and ‘*dehydration*’ were treated as fixed factors, while the field replicate was considered a random factor. Consequently, data were analysed using a linear mixed-effects model (mixed-model ANOVA), with field replicate included as a random effect to account for the correlation among measurements derived from the same biological replicate. Statistical analysis was carried out using Minitab Statistical Software version 19 (Minitab LLC, State College, PA, USA). For both the one-way and two-way ANOVAs, two separate tests were conducted: the first using data expressed on a fresh-weight basis, and the second using data expressed on a dry-weight basis. Prior to ANOVA, the normality of the residuals was assessed using the Shapiro–Wilk test, and homoscedasticity was evaluated using Bartlett’s test. Means were compared using Tukey’s test at a 95% confidence level. A Pearson’s correlation analysis (correlation heatmap) was conducted using Minitab Statistical Software version 19, LLC, to assess the relationships between the physicochemical and antioxidant traits. In this instance, two separate tests were conducted: the first using data expressed on a fresh-weight basis, and the second using data expressed on a dry-weight basis.

Principal component analysis (PCA), a multivariate statistical technique, was employed to simplify the dataset and identify the primary sources of variation among the measured variables. These variables followed a normal distribution, thereby fulfilling the statistical assumptions required for PCA. The analysis was performed using Minitab Statistical Software (Version 19, Minitab, LLC). Given that the variables were measured in different units and scales, they were standardised prior to PCA to eliminate the effects of varying measurement units and scales. As for the ANOVA and Pearson’s correlation analyses, two separate PCA were conducted: one using data expressed on a fresh-weight basis, and the other using data expressed on a dry-weight basis. A biplot was created for each PCA, combining a loading plot and a score plot for the first two principal components.

## 3. Results

### 3.1. Effects of Cultivar on the Physicochemical and Antioxidant Traits in the Fresh Product (on Both Fresh and Dry-Weight Basis)

The effects of ‘*cultivar*’ on the physicochemical and antioxidant characteristics of the fruit examined in this study are shown in Table 1 and Table 2.

Total solids (TS) and total soluble solids (TSS), averaging 10.18 g 100 g^−1^ FW and 7.43 °Brix, respectively, across the three cultivars, did not vary significantly with tomato type (*p* > 0.05) (Table 1). In contrast, clear genetic differences were observed for all other physicochemical characteristics and antioxidants. Total acidity (TA) was the lowest (0.25 g citric a. 100 g^−1^ FW) in the local long shelf-life tomato landrace. The Pachino-type cultivar exhibited the highest reducing sugars content (RS, 3.31 g 100 g^−1^ FW). However, when sweetness was expressed as the TSS/TA ratio (SAR), the local ‘Vulcano’ (29.09) stood out positively compared to the other two types. TA, but not TSS, correlated significantly to SAR (*r* = 0.96 ***. Among antioxidants, vitamin C (AscA), averaging 18.36 mg ascorbic a. 100 g^−1^ FW, was significantly higher in ‘Febo’ and ‘Paskualeto’ (>20 mg in both), and total phenols (TP), averaging 76.57 mg GAE 100 g^−1^ FW, were maximised in ‘Paskualeto’ (87.51 mg, *p* < 0.001). Interestingly, maximum flavonoid (Flav) content (>35 mg RE 100 g^−1^ FW) did not correspond with the highest TP content in ‘Paskualeto’, whose flavonoid content did not reach 30 mg. Lycopene content (Lyco) averaged 5.17 mg 100 g^−1^ FW and peaked in the local landrace ‘Vulcano’ (8.11 mg, *p* < 0.001). Antioxidant activity (AA) averaged 1.37 μmol TE g^−1^ FW and did not vary with the cultivar. Among all antioxidants, a significant positive correlation with AA was found only for TP content (*r* = 0.862 **).

When quality parameters were examined on a dry-weight basis, different results were observed for most of these. TSS showed no significant differences among the cultivars, whereas TA—as previously reported—was lower in the local landrace (Table 2).

All cultivars exhibited the same RS content, whereas SAR was maximised in the long shelf-life tomato ‘Vulcano’. The latter showed the lowest AscA content, whereas TP and Flav levels did not vary according to cultivar. Similar to the observations for fresh weight, Lyco content was higher in the local tomato, and AA showed no variation among the cultivars. As reported for data expressed on a fresh-weight basis, AA was highly correlated and positively with TP (*r* = 0.887 **). A slight but significant positive correlation between AA and Flav content was also found (*r* = 0.555 *).

### 3.2. Effect of Dehydration on the Physicochemical Traits (on a Fresh-Weight Basis)

Selected physicochemical traits were measured in fresh and semi-dry tomato samples.

**Figure 1:**
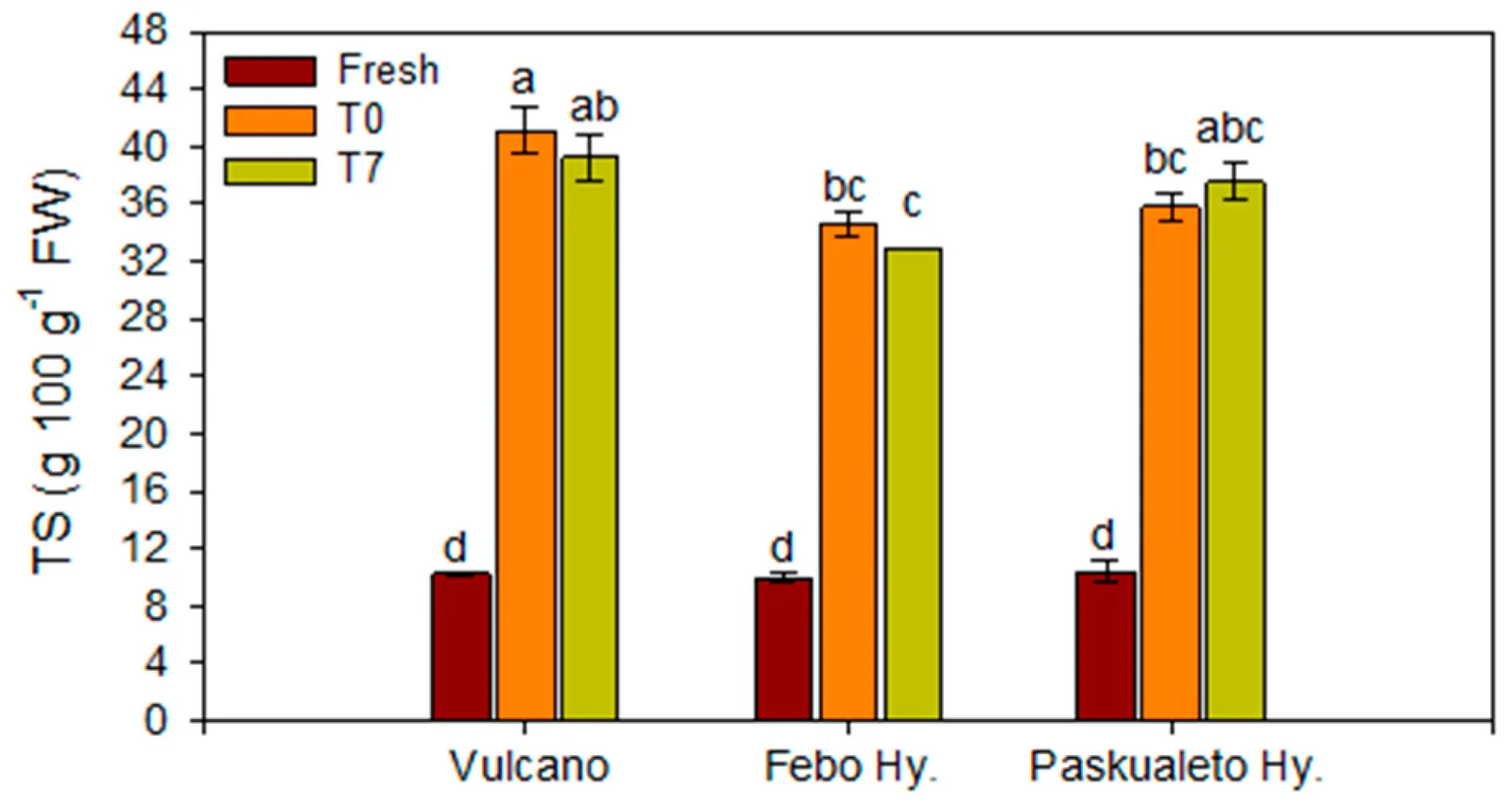
Effect of ‘*dehydration* × *cultivar*’ (*D* × *C*) interaction on total solids (TS) in tomato. Values are the means ± standard error (*SE*) (*n* = 3). Different letters indicate significance at *p* ≤ 0.05 (Tukey’s test).

TS content increased significantly after dehydration, to 37.17 g 100 g^−1^, and did not change after a week of cold storage (Table 3). Among the cultivars, the long shelf-life type exhibited the highest TS content (30.19 g 100 g^−1^), which was 17 and 9% higher than those of ‘Febo Hy.’ and ‘Paskualeto Hy.’, respectively. However, ‘*cultivar*’ contributed only 2% of the total variance (Table 4). A significant ‘*C* × *D*’ (*p* < 0.05) interaction indicates that the effect of dehydration varied among cultivars. In fact, they had the same TS in the fresh product (~10 g 100 g^−1^ FW), but after dehydration, TS peaked significantly in the local landrace, exceeding 40 g 100 g^−1^ (Figure 1). In this tomato, the content was unchanged after a week of cold storage. Both miniplum and Pachino-type tomatoes did not differ in TS content in the semi-dry product, just after dehydration and one week later.

**Figure 2:**
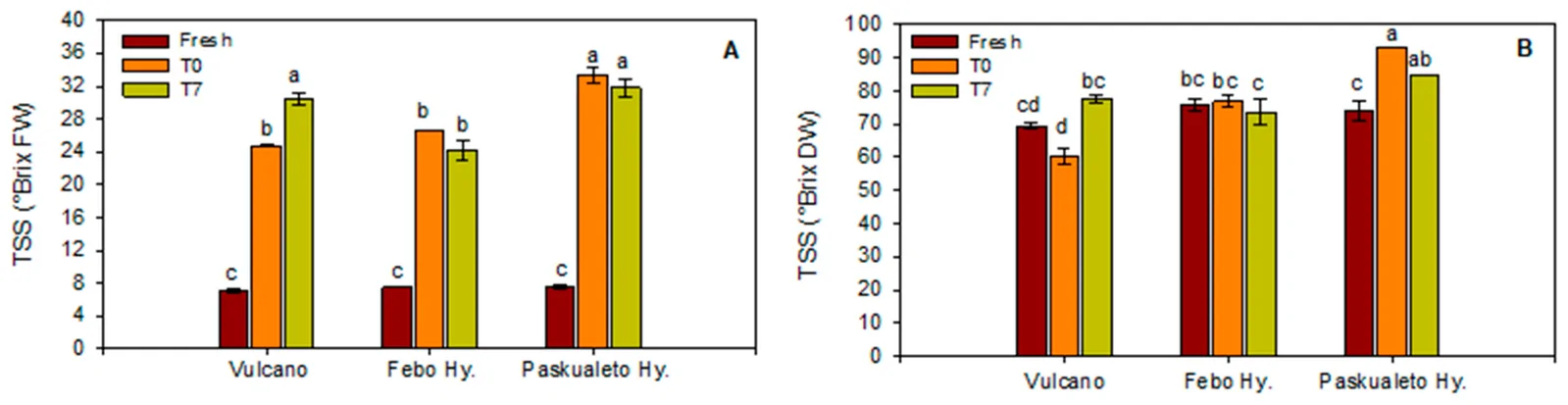
Effect of ‘*dehydration* × *cultivar*’ (*D* × *C*) interaction on the total soluble solids (TSS) in tomato ((**A**): on a fresh-weight basis; (**B**): on a dry-weight basis). Values are the means ± standard error (*SE*) (*n* = 3). Different letters indicate significance at *p* ≤ 0.05 (Tukey’s test).

Dehydration significantly increased the TSS content*,* which rose from 7.43 to 28.22 °Brix (*D*, *p* < 0.001). This level was maintained in the semi-dry product after one week of cold storage (T7). As for TS, the ‘*cultivar*’ contribution to total variance was minimal (4%). Across the dehydration treatments, ‘Paskualeto’ (Pachino-type) had the highest TSS content (>24 °Brix). The two factors interacted significantly for this physicochemical parameter (*p* < 0.001). In the fresh product, the three cultivars exhibited the same TSS; however, after drying (T0), the semi-dry product from the ‘Paskualeto’ cultivar showed a higher content (>30 g 100 g^−1^) compared to ‘Febo’ and ‘Vulcano’—a value that remained unchanged after one week (Figure 2A). Unlike ‘Febo’ and ‘Paskualeto’, the old landrace ‘Vulcano’ experienced a further increase in TSS at T7, to the same level as the Pachino-type. When TSS content was expressed on a dry-weight basis, different results were obtained. Indeed, the contribution of ‘*cultivar*’ rose to 62%, indicating that the tomato’s water content was somewhat masking the actual genetic differences in this physicochemical trait. ‘Paskualeto’ had overall higher TSS (>83 °Brix). Furthermore, variations in TSS content were no longer evident after the tomatoes were dehydrated. This was particularly the case for ‘Vulcano’ and ‘Febo’, whose TSS content was unaffected by the drying treatment (*C* × *D*, *p* < 0.001).

**Figure 3:**
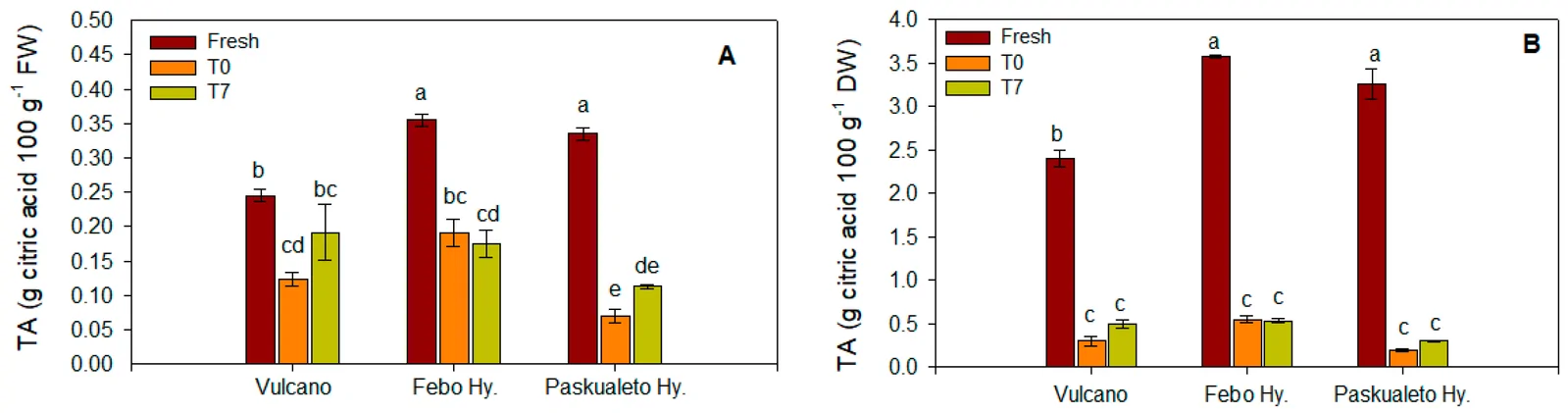
Effect of ‘*dehydration* × *cultivar*’ (*D* × *C*) interaction on the titratable acidity (TA) in tomato ((**A**): on a fresh-weight basis; (**B**): on a dry-weight basis). Values are the means ± standard error (*SE*) (*n* = 3). Different letters indicate significance at *p* ≤ 0.05 (Tukey’s test).

*TA* of tomatoes was 0.31 g citric a. 100 g^−1^ FW, and decreased significantly to 0.13 g 100 g^−1^ after dehydration (T0) (*D*, *p* < 0.001). A 23% increase in TA was measured after a week (T7). The greatest titratable acidity was measured in the miniplum ‘Febo’ (0.24 g 100 g^−1^). ‘*Dehydration*’ (50%), more than ‘*cultivar*’ (7%), contributed to the total variance in TA of tomatoes. A significant ‘*D* × *C*’ (*p* < 0.001) in the ANOVA indicated that the effect of the dehydration treatment differed among cultivars. In particular, TA of fresh samples was significantly higher in the two hybrids, but following heat treatment, the content decreased to a greater extent (−81%) in the Pachino-type (Figure 3A).

**Figure 4:**
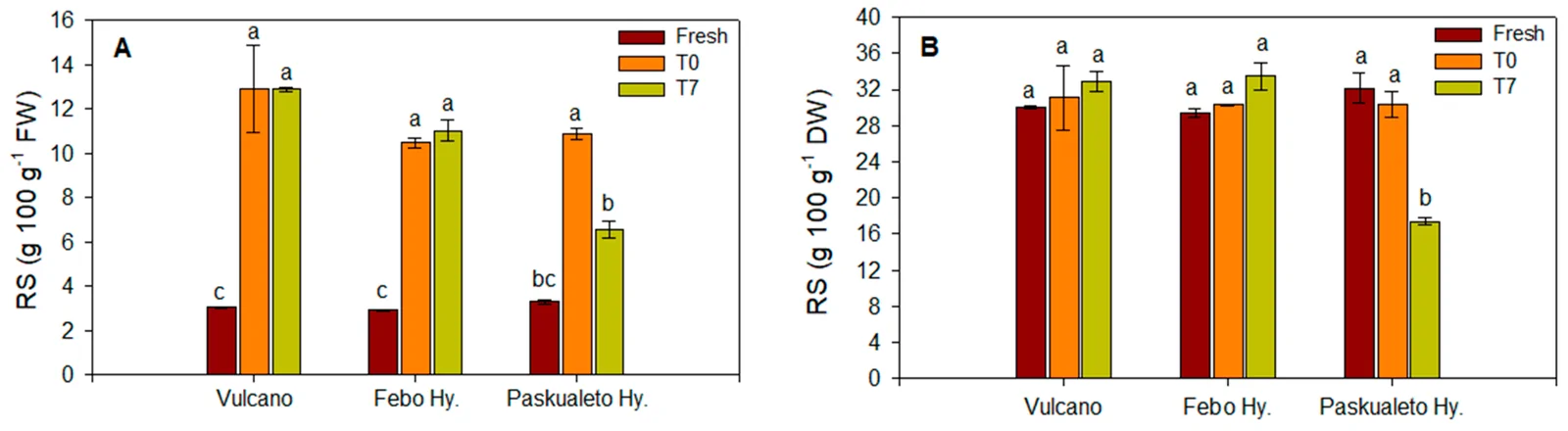
Effect of ‘*dehydration* × *cultivar*’ (*D* × *C*) interaction on the reducing sugars (RS) in tomato ((**A**): on a fresh-weight basis; (**B**): on a dry-weight basis). Values are the means ± standard error (*SE*) (*n* = 3). Different letters indicate significance at *p* ≤ 0.05 (Tukey’s test).

*RS* content was 3.10 g 100 g^−1^ FW in the fresh samples. After dehydration, this level increased significantly, to 11.41 g 100 g^−1^, and remained stable thereafter. Among the cultivars, the richest in RS was the local landrace ‘Vulcano’ (9.61 g 100 g^−1^). As reported for the physicochemical traits previously discussed, ‘*D*’ and ‘*C*’ significantly interacted for this trait (*p* < 0.001) (Figure 4A). The cultivars did not differ in RS content in the fresh and just-dehydrated (T0) samples; however, one week later (T7), the RS content of semi-dry tomatoes was significantly reduced (−40%) in the Pachino-type, whereas it remained unchanged in the other two types of tomatoes.

**Figure 5:**
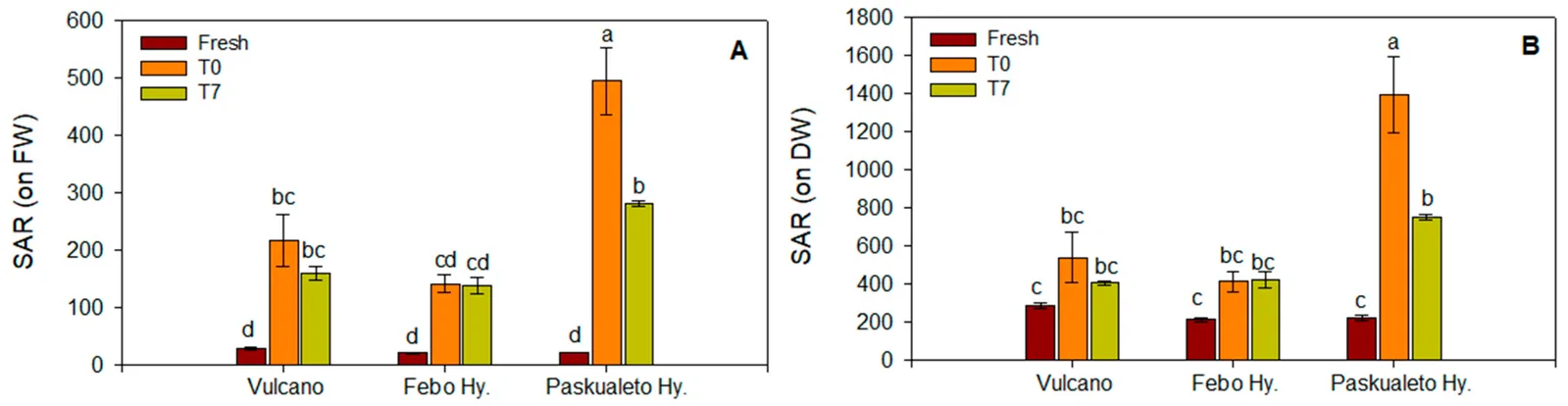
Effect of ‘*dehydration* × *cultivar*’ (*D* × *C*) interaction on the ratio TSS/TA (SAR) in tomato ((**A**): on a fresh-weight basis; (**B**): on a dry-weight basis). Values are the means ± standard error (*SE*) (*n* = 3). Different letters indicate significance at *p* ≤ 0.05 (Tukey’s test).

The ratio of TSS/TA (SAR), another physicochemical index of sweetness, increased significantly after dehydration (from 24.38 to 284.72). Cold storage reduced the SAR of semi-dry tomatoes by 32% at T7. Across the dehydration treatments, ‘Paskualeto’ exhibited higher SAR than ‘Febo’ and ‘Vulcano’. The significant combination ‘*D* × *C*’ (*p* < 0.001) indicated that the dehydration effect differed among the cultivars. At T0, the semi-dry ‘Paskualeto’ had significantly higher SAR than ‘Febo’ and ‘Vulcano’, but after one week, SAR decreased to the same level as that of ‘Vulcano’ (Figure 5A).

### 3.3. Effect of Dehydration on the Physicochemical Traits (on a Dry-Weight Basis)

Different results were obtained when the selected physicochemical traits were expressed on a dry-weight basis.

In TSS, the contribution of the ‘*cultivar*’ to total variance increased to 62% (Table 5), indicating that tomato water content partially masked the underlying genetic differences in this physicochemical trait. Overall, the Pachino-type had the highest TSS (>83 °Brix) (Table 6). Unlike when expressed on a fresh-weight basis, variations in TSS content after the tomatoes were dehydrated were less evident (*p* < 0.05). This was particularly the case for ‘Vulcano’ and ‘Febo’, whose TSS content was unaffected by the drying treatment (*C* × *D*, *p* < 0.001) (Figure 2B).

TA content was drastically reduced (−89%) by the dehydration treatment, and remained constant one week later (T7). ‘Febo’ had the highest TA (1.55 g citric a. 100 g^−1^), followed by ‘Paskualeto’ and ‘Vulcano’. The ‘*D* × *C*’ interaction (*p* < 0.001) was significant in this case as well. Unlike when the content was expressed on a fresh-weight basis, all cultivars exhibited the same TA content after dehydration, and one week later (Figure 3B).

RS content, expressed on a dry-weight basis, did not change after dehydration and remained constant thereafter. ‘Paskualeto’ was the poorest in RS (26.64 g 100 g^−1^). The two factors interacted significantly on the RS content (*p* < 0.001). No differences were observed among cultivars either in fresh tomatoes or in the semi-dry, just-dehydrated product; a week later, the RS content of the ‘Paskualeto’ semi-dry was reduced significantly by 43% (Figure 4B).

The TSS/TA ratio (SAR), another physicochemical index of sweetness, increased significantly after dehydration (from 240.39 to 781.05). One week later, the SAR value decreased by 32%. Across the dehydration treatments, the Pachino-type recorded the highest SAR value. The significant combination ‘*D* × *C*’ (*p* < 0.001) indicated that the dehydration effect differed among the cultivars. At T0, the semi-dry ‘Paskualeto’ had significantly higher SAR than ‘Febo’ and ‘Vulcano’, but after one week, the ratio decreased to the same level as that of the other cultivars (Figure 5B).

### 3.4. Effect of Dehydration on the Antioxidant Traits (on a Fresh-Weight Basis)

Selected antioxidant traits were measured in fresh and semi-dry tomatoes. Among all the traits, the ‘*cultivar*’ contributed the most (85%) to the total variance of lycopene content (Table 7).

**Figure 6:**
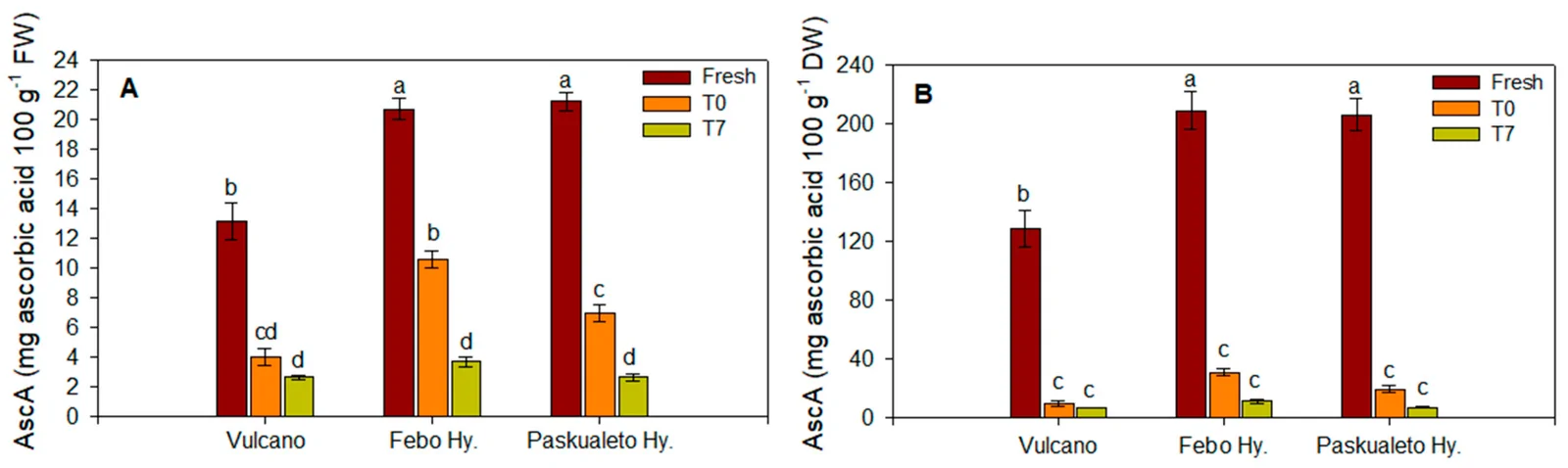
Effect of ‘*dehydration* × *cultivar*’ (*D* × *C*) interaction on the ascorbic acid content (AscA) in tomato ((**A**): on a fresh-weight basis; (**B**): on a dry-weight basis). Values are the means ± standard error (*SE*) (*n* = 3). Different letters indicate significance at *p* ≤ 0.05 (Tukey’s test).

Dehydration caused a substantial decrease in AscA content (−61%) (Table 8). A further decrease (−58%) occurred in cold-stored semi-dry tomatoes. Among the cultivars, the lowest levels of AscA were found in the local landrace of long shelf-life tomato (6.61 mg 100 g^−1^ ascorbic acid). The miniplum ‘Febo’ was the richest in AscA (11.67 mg). The two experimental factors interacted significantly on this antioxidant trait (*D* × *C*, *p* < 0.001). The three cultivars exhibited the same content in the fresh tomatoes. After dehydration, the miniplum ‘Febo’ maintained a higher AscA level than ‘Paskualeto’ and ‘Vulcano’. However, one week later, differences among the cultivars were no longer visible (Figure 6A).

**Figure 7:**
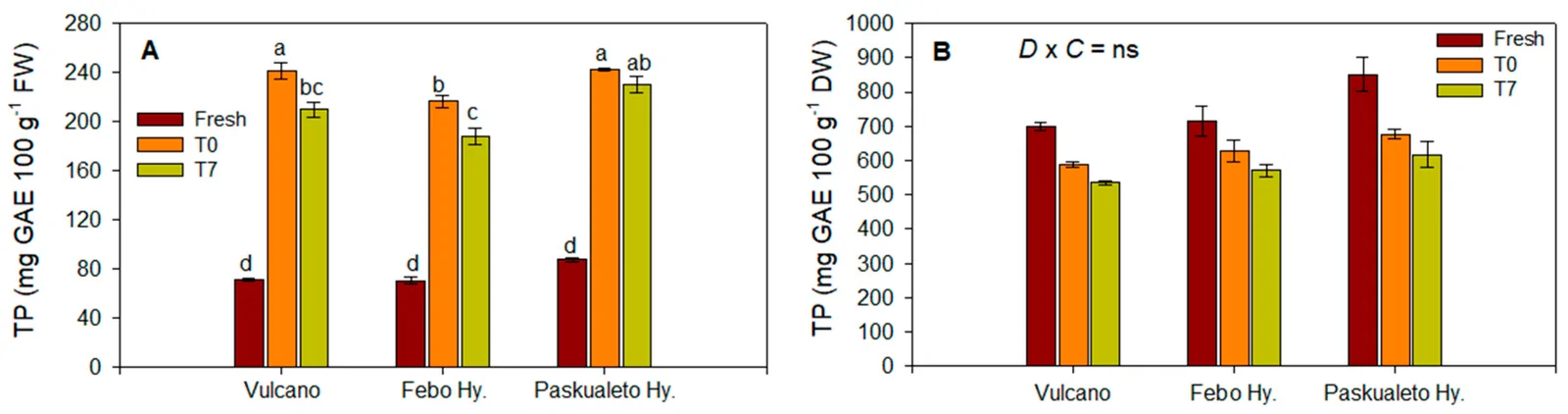
Effect of ‘*dehydration* × *cultivar*’ (*D* × *C*) interaction on the total phenol content (TP) in tomato ((**A**): on a fresh-weight basis; (**B**): on a dry-weight basis). Values are the means ± standard error (*SE*) (*n* = 3). Different letters indicate significance at *p* ≤ 0.05 (Tukey’s test); ns: not significant.

The *TP* content increased significantly after dehydration (from 76.6 to 233.3 mg GAE 100 g^−1^ FW). However, it decreased by 10% in the cold-stored semi-dry tomatoes. Across the dehydration treatments, the two hybrids ‘Paskualeto’ and ‘Febo’ exhibited the highest (186.6 mg) and the lowest (158.4 mg) content, respectively. The ANOVA revealed a slight but significant interaction between the two factors (*p* < 0.001). In the semi-dry product, TP content increased significantly more in ‘Vulcano’ and ‘Paskualeto’ than in ‘Febo’, but one week later, TP content decreased significantly in ‘Vulcano’, approaching that in ‘Febo’ (Figure 7A).

**Figure 8:**
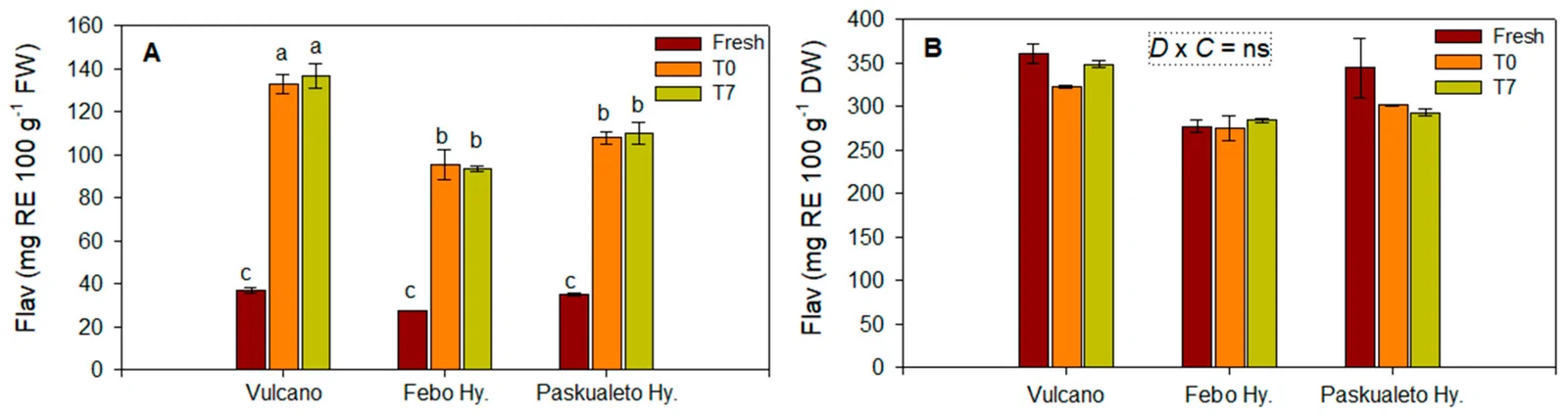
Effect of ‘*dehydration* × *cultivar*’ (*D* × *C*) interaction on the flavonoid content (Flav) in tomato ((**A**): on a fresh-weight basis; (**B**): on a dry-weight basis). Values are the means ± standard error (*SE*) (*n* = 3). Different letters indicate significance at *p* ≤ 0.05 (Tukey’s test); ns: not significant.

Dehydration of tomatoes resulted in a significant increase in flavonoid content (Flav) (from 33.2 to 111.9 mg RE 100 g^−1^ FW). This content was maintained in cold-stored semi-dry tomatoes. Unlike TP, the local landrace ‘Vulcano’ was the richest in these antioxidants (102.0 mg). The significant ‘*D* × *C*’ interaction (*p* < 0.01) showed that the tomatoes did not differ in Flav content in the fresh product, but in the semi-dry product, ‘Vulcano’ maintained higher Flav contents than the two hybrids, both after dehydration and one week later (Figure 8A).

**Figure 9:**
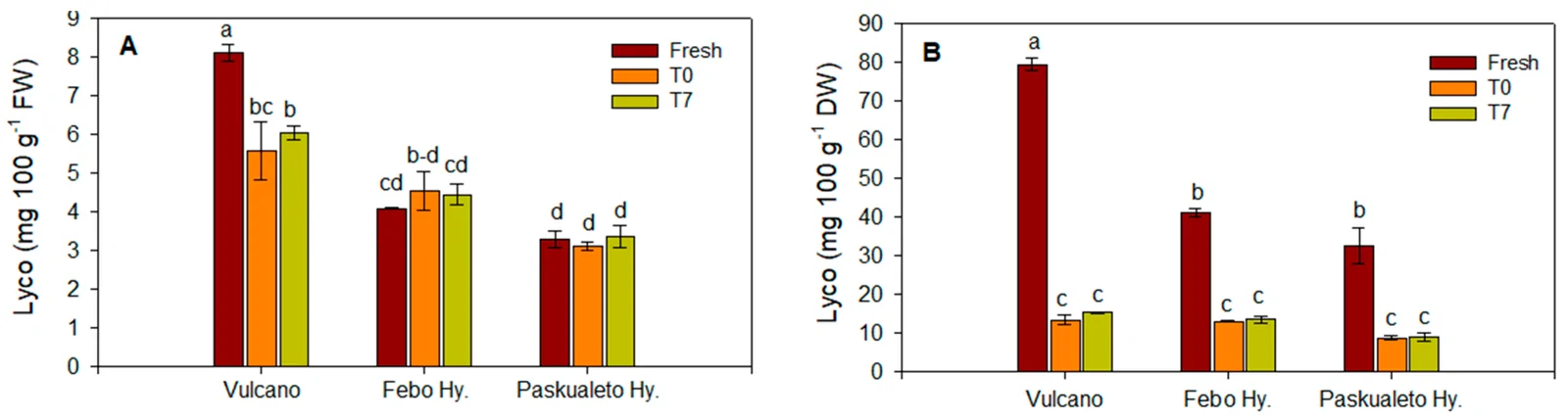
Effect of ‘*dehydration* × *cultivar*’ (*D* × *C*) interaction on the lycopene content (Lyco) in tomato ((**A**): on a fresh-weight basis; (**B**): on a dry-weight basis). Values are the means ± standard error (*SE*) (*n* = 3). Different letters indicate significance at *p* ≤ 0.05 (Tukey’s test).

Unlike the other antioxidants, ‘*dehydration*’ contributed less to the total variance in lycopene content (Lyco). Across cultivars, semi-dry tomatoes had a 10% lower Lyco content (from 5.16 to 4.62 mg 100 g^−1^) than fresh tomatoes. This content remained unchanged in semi-dry tomatoes analysed one week later. Among cultivars, Lyco was significantly higher in ‘Vulcano’ (6.57 mg). However, unlike the two hybrids, the latter experienced a significant reduction in this antioxidant following dehydration, although it still maintained a higher level (>5 mg) than ‘Paskualeto’ and ‘Febo’ (*D* × *C*, *p* < 0.001) (Figure 9A).

**Figure 10:**
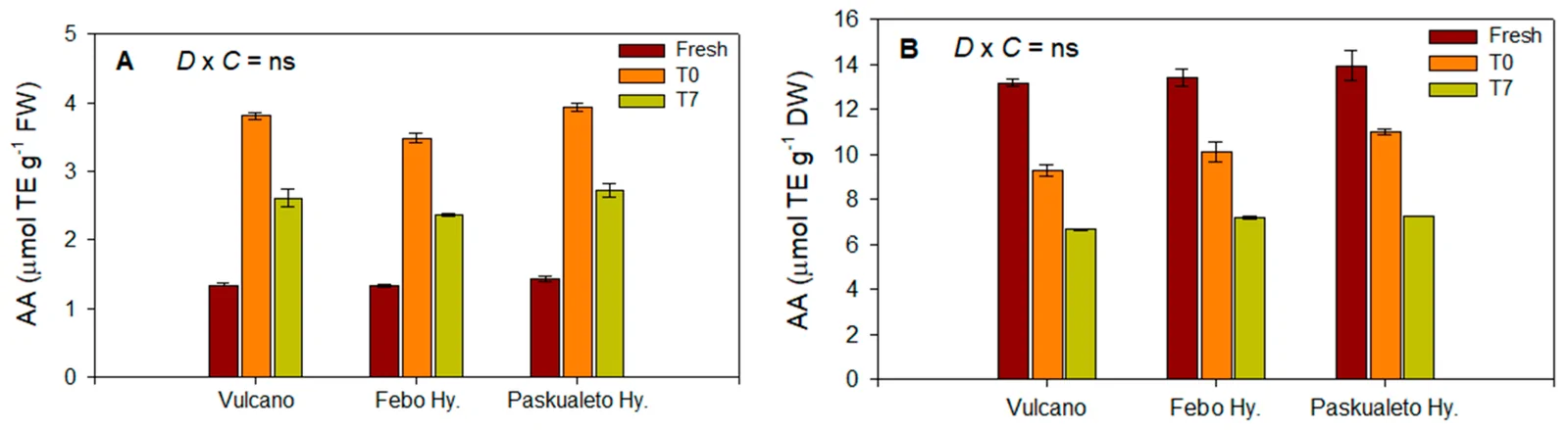
Effect of ‘*dehydration* × *cultivar*’ (*D* × *C*) interaction on the antioxidant activity (AA) in tomato ((**A**): on a fresh-weight basis; (**B**): on a dry-weight basis). Values are the means ± standard error (*SE*) (*n* = 3). ns: not significant.

The antioxidant activity (AA) was significantly higher in ‘Vulcano and ‘Paskualeto’ compared to ‘Febo’. Averaged across the cultivars, AA more than doubled in semi-dry tomatoes immediately after dehydration (T0), rising from 1.37 to 3.74 μmol TE g^−1^ FW. After one week of cold storage, a significant decrease in AA (−31%) was observed in the semi-dry product. This result was consistent across all tomato cultivars (*D* × *C*, *p* > 0.05) (Figure 10A).

### 3.5. Effect of Dehydration on the Antioxidant Traits (on a Dry-Weight Basis)

As with the physicochemical traits, the antioxidant traits were also expressed on a dry-weight basis. The ‘*dehydration*’ treatment, more than the ‘*cultivar*’, contributed to the total variance in most of the antioxidant properties (Table 9).

The ascorbic acid (AscA) content was markedly reduced by dehydration, from 181.4 to 20.1 mg 100 g^−1^ DW (Table 10). One week later, a further decrease (−58%) occurred in cold-stored semi-dry tomatoes. Among the cultivars, the local landrace of long shelf-life tomato exhibited the lowest AscA content (48.5 mg). The two experimental factors interacted significantly on this trait (*D* × *C*, *p* < 0.001); unlike the observations for fresh tomatoes—where the content in the ‘Paskualeto’ and ‘Febo’ cultivars was significantly higher than in ‘Vulcano’—no differences emerged among the three cultivars in the semi-dry product, at either time T0 or T7 (Figure 6B).

As observed for AscA, total phenol (TP) content decreased following dehydration (T0, −16%). Subsequently, this content remained unchanged in cold-stored semi-dry tomatoes (T7). The cultivar with the highest TP content was ‘Paskualeto’ (*C*, *p* < 0.001), at both T0 and T7 (*D* × *C*, *p* > 0.05) (Figure 7B).

A trend similar to that of TP was observed in flavonoid (Flav) content, which decreased significantly following dehydration (−8% at T0) and then remained unchanged one week later (T7). The ‘Vulcano’ landrace and ‘Febo’ miniplum cultivar had the highest and the lowest Flav content, respectively. As reported for TP, the two experimental factors did not significantly interact on this trait (*D × C*, *p* > 0.05) (Figure 8B).

Like other antioxidants, lycopene (Lyco) was negatively affected by dehydration, with its content decreasing by 77%. This value remained unchanged in the semi-dry product after one week of cold storage. ‘Vulcano’ was much richer in Lyco than ‘Paskualeto’ and ‘Febo’. A significant ‘*D × C*’ (*p* < 0.001) showed a different cultivar response to the dehydration treatment: the greater Lyco content in fresh tomatoes of ‘Vulcano’ compared with ‘Paskualeto’ and ‘Febo’ was not maintained in semi-dry tomatoes, at both T0 and T7 (Figure 9B). The significant decrease in the content of all antioxidants following dehydration resulted in a significant reduction in antioxidant activity (AA) (from 13.5 to 10.11 μmol TE g^−1^ DW) in semi-dry tomatoes. AA decreased further (−30%) in the semi-dry product after one week. ANOVA revealed no significant ‘*D × C*’ interaction (Figure 10B). Indeed, a similar response of the cultivars to the dehydration treatment was observed.

### 3.6. Pearson’s Correlation Analysis

A Pearson’s correlation test was conducted to examine correlations among the physicochemical and antioxidant traits of tomatoes, considering the pooled dataset (fresh and semi-dry tomatoes at T0 and T7).

**Figure 11:**
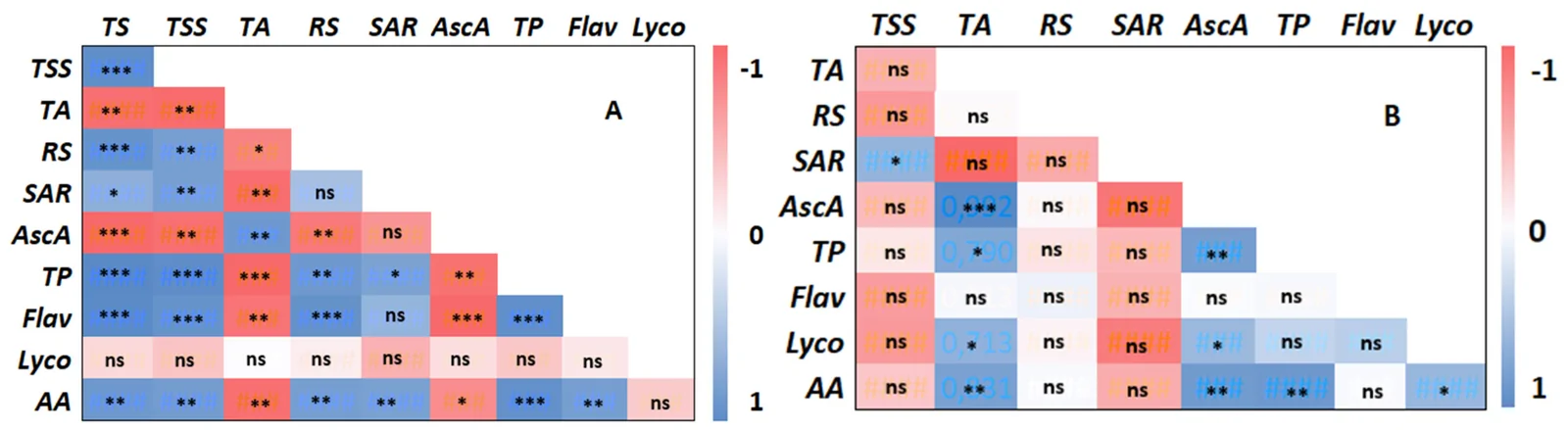
Heatmap of Pearson’s correlation matrix for the studied traits in tomato ((**A**): on a fresh-weight basis; (**B**): on a dry-weight basis). Different abbreviations used in the figures are as follows: TS: total solids; TSS: total soluble solids; TA: titratable acidity; RS: reducing sugars; SAR: TSS/TA. AscA: ascorbic acid; TP: total phenols; Flav: flavonoids; Lyco: lycopene; AA: antioxidant activity. *, **, and *** indicate significance at *p* ≤ 0.05, 0.01, and 0.001, respectively; ns = not significant.

The correlation analysis (data on a fresh-weight basis) revealed several strong associations among the measured variables. TS showed very strong positive correlations with TSS (*r* = 0.951 ***), RS (*r* = 0.911 ***), TP (*r* = 0.981 ***), Flav (*r* = 0.980 ***), and AA (*r* = 0.871 **), and strong negative correlations with TA (*r* = −0.864 **) and AscA (*r* = −0.905 ***) (Figure 11A). Similarly, TSS was positively correlated with RS (*r* = 0.818 **), SAR (*r* = 0.821 **), TP (*r* = 0.963 ***), Flav (*r* = 0.915 ***), and AA (*r* = 0.843 **), but negatively correlated with TA (*r* = −0.886 **) and AscA (*r* = −0.860 **).

TA exhibited negative correlations with RS (r = −0.724 *), SAR (*r* = −0.878 **), TP (*r* = −0.903 ***), Flav (*r* = −0.830 **), and AA (*r* = −0.855 **), whereas it was positively associated with AscA (*r* = 0.869 **). It is interesting to note that AscA was negatively correlated with quality parameters related to antioxidant properties, including TP (*r* = −0.848 **), Flav (*r* = −0.908 ***), and AA (*r* = −0.666 *), and among physicochemical quality parameters, with RS (*r* = −0.799 **). TP and Flav were strongly correlated (*r* = 0.943 ***). Both antioxidants also showed strong positive correlations with AA (*r* = 0.929 *** and *r* = 0.827 **, respectively). In contrast, Lyco exhibited no correlation with the variables examined.

Different results were obtained when data were considered on a dry-weight basis. TSS showed no correlation with the other variables, except SAR (*r* = 0.695 *) (Figure 11B). TA exhibited a very high positive correlation with AscA (*r* = 0.992 ***). Positive correlations were also observed with TP (*r* = 0.790 *), Lyco (*r* = 0.713 *), and AA (*r* = 0.831 **). RS and SAR did not correlate with any of the examined traits. AscA was found to be strongly and positively correlated with AA (*r* = 0.877 **), TP (*r* = 0.854**), and Lyco (*r* = 0.666*). TP showed a very strong positive association with AA (*r* = 0.889 **), whereas Lyco showed a moderate positive correlation with it (*r* = 0.675 *).

### 3.7. PCA Component Analysis (Data on Fresh-Weight Basis)

A principal component analysis (PCA) was conducted to evaluate the effects of the experimental factors (‘*dehydration*’ and ‘*cultivar*’) on the physicochemical and antioxidant traits examined, identifying potential groupings (clusters) among the treatments. The length of the vectors provided information on the contribution of each trait to the observed differentiation among treatments: longer vectors indicate traits strongly correlated with the principal components, which consequently contribute more substantially to sample discrimination. Two separate PCA analyses were conducted: the first using fresh-weight-based data and the second using dry-weight-based data.

#### 3.7.1. PCA Component Analysis (Data on a Fresh-Weight Basis)

**Figure 12:**
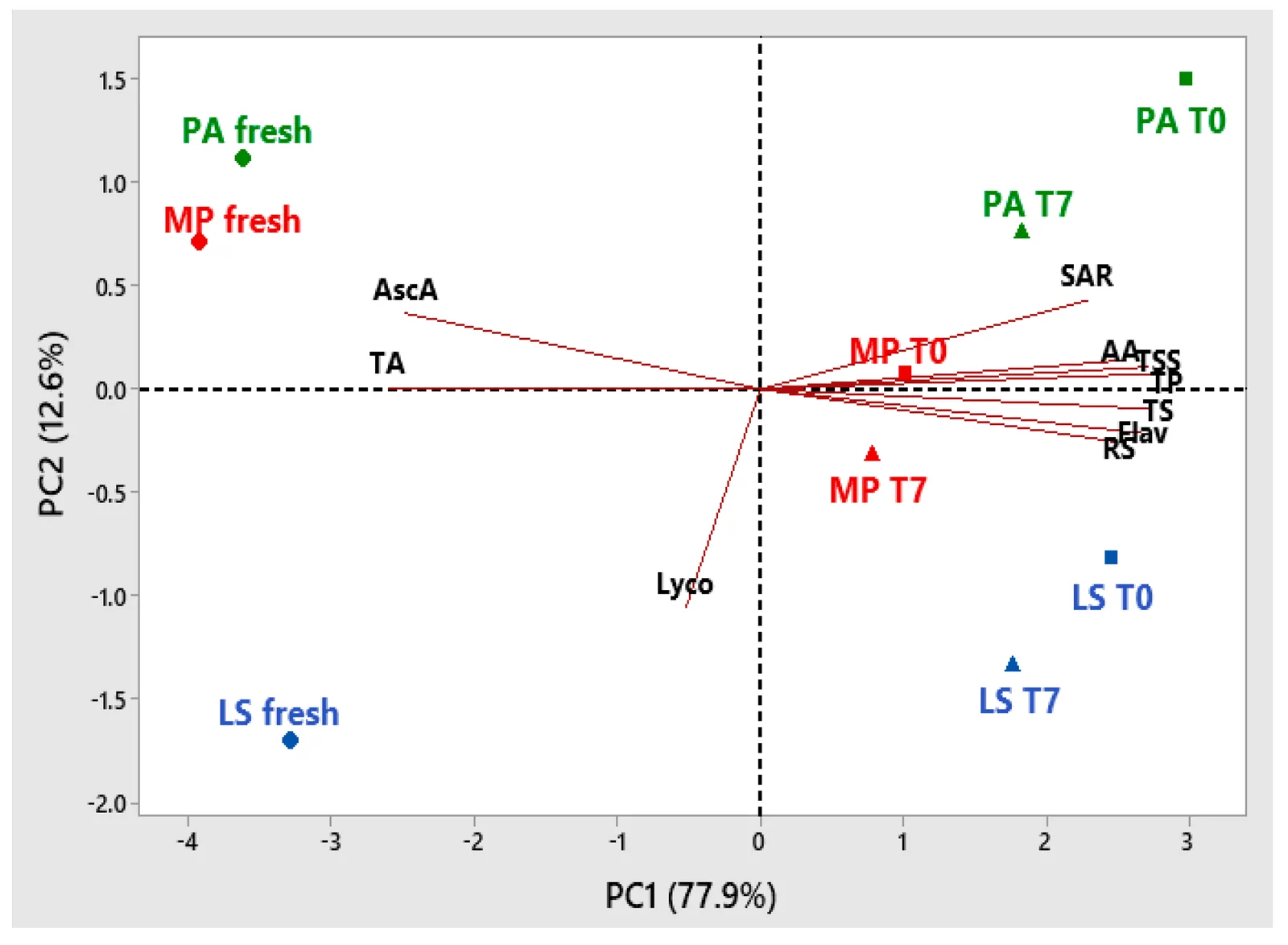
PCA biplot of loadings and scores for physicochemical (TS: totals solids; TSS: total soluble solids; TA: titratable acidity; RS: reducing sugars; SAR: TSS/TA) and antioxidant traits (AscA: ascorbic acid; TP: total phenols; Flav: flavonoids; Lyco: lycopene; AA: antioxidant activity) in tomato (on a fresh-weight basis), according to ‘*dehydration*’ (fresh: fresh tomatoes; T0: semi-dry tomatoes just after dehydration; T7: semi-dry tomatoes after a week of cold storage) and ‘*cultivar*’ (MP: ‘Febo Hy.’ miniplum; PA: ‘Paskualeto Hy.’ of Pachino tomato; LS: ‘Vulcano’ of long shelf-life tomato).

PCA (data on a fresh-weight basis) identified two factors with eigenvalue > 1 (PC1 and PC2), which together explained 90.5% of total variance (Figure 12). PCA revealed a clear separation of samples according to treatment along PC1 and PC2.

Loading analysis showed that most quality traits, including SAR, TS, TSS, RS, TP, Flav, and AA, were primarily aligned with PC1; furthermore, their relatively long vectors indicate a substantial contribution to the sample discrimination (Table 11).

AscA and TA were both oriented toward the negative side of PC1, indicating their association with fresh tomatoes. Because PC1 explained most of the variance (77.9%), both traits contributed substantially to the discrimination between fresh and semi-dry tomatoes. The similar direction of their vectors confirms the positive correlation between these traits (see Section 3.5). Conversely, Lyco showed a relatively short vector, oriented toward the negative side of PC2. Therefore, Lyco contributed only marginally to the overall discrimination between fresh and semi-dry tomatoes.

Fresh tomatoes (LS fresh, MP fresh, PA fresh) clustered on the negative side of PC1. The loading vectors for TA and AscA pointed toward the same quadrant as PA fresh and MP fresh, indicating that these tomatoes were characterised by high acidity and ascorbic acid content. Lyco was closely associated with fresh LS tomato samples, which were positioned along PC2.

All semi-dry tomatoes were all positioned on the opposite side of PC1. Pachino-type (PA) semi-dry tomatoes, at both T0 and T7, were located in the upper-right quadrant. They were positively associated with physicochemical quality-related traits such as TSS and SAR; the latter also showed a positive correlation with AA. Conversely, semi-dry tomatoes from the local long shelf-life tomato (LS), at both T0 and T7, were positioned in the lower-right quadrant, near the vectors for TS, RS, and Flav, highlighting a strong relationship with these quality traits. Semi-dry tomatoes from the miniplum ‘Febo’ (MP), at both T0 and T7, were placed in an intermediate position, suggesting a weaker association with the examined quality traits.

#### 3.7.2. PCA Component Analysis (Data on a Dry-Weight Basis)

**Figure 13:**
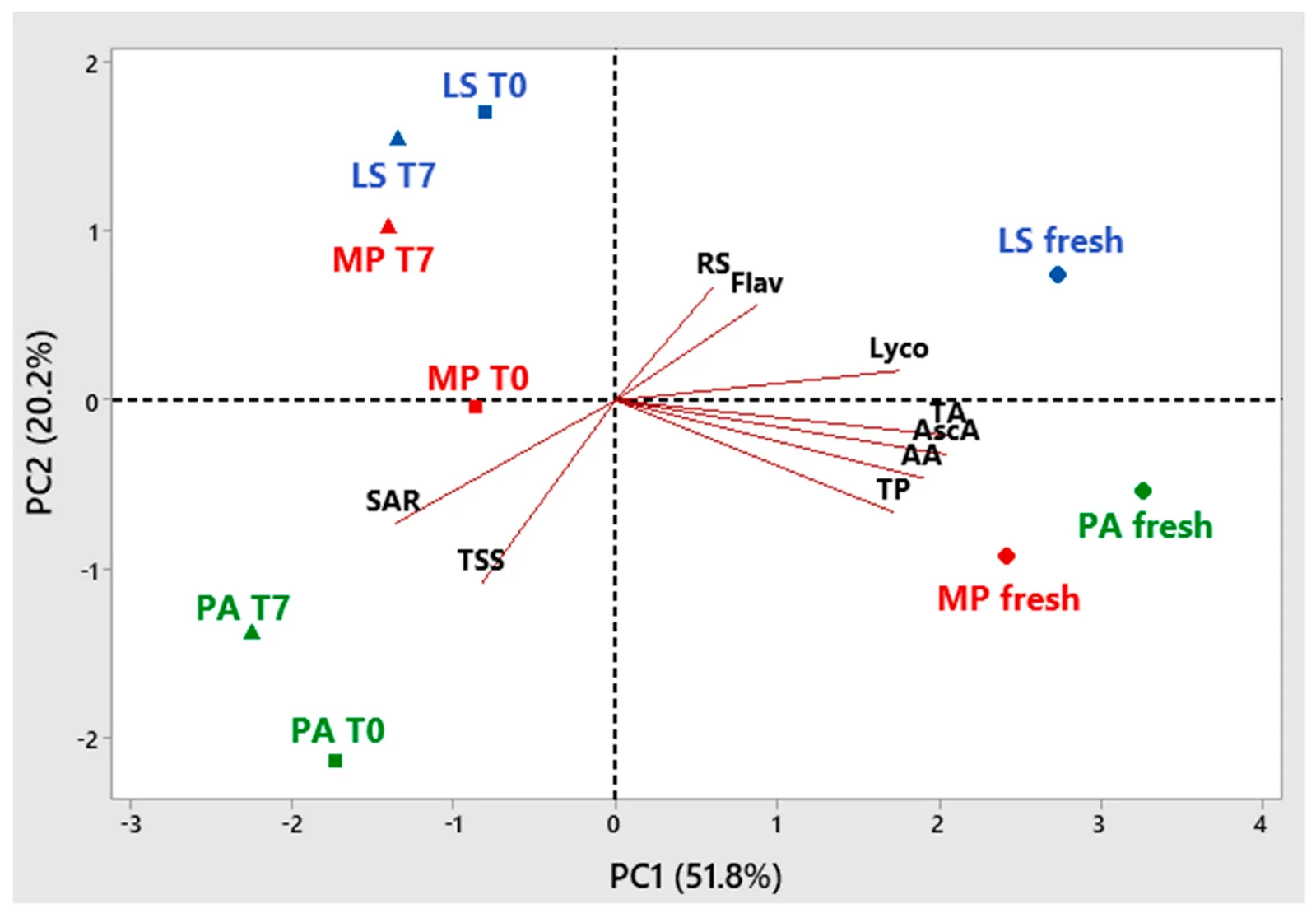
PCA biplot of loadings and scores for physicochemical (TS: totals solids; TSS: total soluble solids; TA: titratable acidity; RS: reducing sugars; SAR: TSS/TA) and antioxidant traits (AscA: ascorbic acid; TP: total phenols; Flav: flavonoids; Lyco: lycopene; AA: antioxidant activity) in tomato (on a dry-weight basis), according to ‘*dehydration*’ (fresh: fresh tomatoes; T0: semi-dry tomatoes just after dehydration; T7: semi-dry tomatoes after a week of cold storage) and ‘*cultivar*’ (MP: ‘Febo Hy.’ miniplum; PA: ‘Paskualeto Hy.’ of Pachino tomato; LS: ‘Vulcano’ of long shelf-life tomato).

PCA (data on a dry-weight basis) explained 72.0% of the total variance, with PC1 accounting for 51.8% and PC2 accounting for 20.2% (Figure 13). In this case, PC1 clearly separated the fresh samples from semi-dry tomatoes at both T0 and T7.

The three fresh tomato samples (LS, MP, and PA) were positioned on the positive side of PC1. They were strongly associated with TA, RS, AscA, TP, Flav, Lyco, and AA—parameters that contributed to their separation from the semi-dry tomatoes. Conversely, the semi-dry tomatoes, at both T0 and T7, were grouped on the negative side of PC1, indicating lower values for variables associated with fresh fruits and, consequently, lower nutritional and phytochemical quality traits compared to fresh tomatoes. PC2 discriminated primarily between the samples based on RS and Flav, which exhibited positive loadings and were oriented towards the upper part of the score plot.

The LS tomatoes at T0 and T7 and semi-dry MP tomatoes at T7 were clustered in the upper-left quadrant of the PCA score plot, exhibiting positive PC2 scores. Their position along the RS and Flav vectors indicated a stronger association with reducing sugars and flavonoids and, consequently, a distinct metabolic profile. Conversely, they were clearly separated from LS fresh samples along PC1 and from semi-dry PA tomatoes along PC2, highlighting the distinct nutritional composition of semi-dry LS tomatoes. The proximity between LS T0 and LS T7 indicated a high degree of similarity between the two time points, suggesting that cold storage had a limited effect on the overall physicochemical profile of the semi-dry LS product.

Although SAR and TSS were negatively correlated with PC1, their strongest contribution was observed along PC2, as indicated by the pronounced downward orientation of their vectors. Therefore, these attributes played a key role in discriminating the tomato samples along PC2, separating the semi-dry samples of the ‘Paskualeto’ cultivar (PA T0 and PA T7)—showing a stronger association with TSS and SAR—from those positioned in the upper part of the score plot. Other quality traits, including Lyco, TA, AA, AscA, and TP, aligned primarily with PC1, only marginally discriminating along PC2.

The MP tomatoes were located in an intermediate position, suggesting a response pattern between those of LS and PA.

Although all fresh tomatoes clustered on the right side of PC1—associated with antioxidant and nutritional traits—their separation along PC2 (with LS in the upper-right quadrant, MP and PA in the lower-right) indicates cultivar-related differences in the relative amount of these compounds.

## 4. Discussion

This study assessed the suitability of a local long shelf-life tomato landrace (‘Vulcano’, LS) for producing a semi-dry product, comparing it with a traditional miniplum cultivar (‘Febo’ Hy., MP) and a Pachino-type cultivar (‘Paskualeto’ Hy., PA). All tomatoes were grown without irrigation after plant establishment, following the traditional method used for local long shelf-life landraces [2,15].

All quality attributes were expressed on both a fresh-weight and a dry-weight basis to evaluate the product’s quality as it is (fresh-weight basis) and to eliminate the influence of sample moisture content (dry-weight basis). In the latter case, differences among samples reflect genuine variations in physicochemical and antioxidant characteristics related to the experimental factors—cultivar and dehydration in this study—allowing for a more accurate comparison between treatments.

### 4.1. Fresh Product

Prior to dehydration, all samples were assessed for their physicochemical and antioxidant qualities. Fresh tomatoes of the LS cultivar exhibited higher SAR values—both on a fresh-weight and dry-weight basis—compared to the two commercial hybrids. The elevated SAR values in local landraces underscore the importance of their physicochemical and nutritional characteristics [23]. Indeed, the high SAR imparts a sweeter taste to LS fruits compared to commercial hybrids, despite their RS content being lower than that of the Pachino-type (PA) tomato, which is renowned worldwide for its exceptional sweetness [14]. SAR is a key quality index that influences perceived flavour and sweetness; variations in SAR are linked to fluctuations in both total soluble solids (TSS) and titratable acidity (TA) [24]. TSS and TA are critical factors in determining tomato flavour, with high and balanced levels of both being desirable [25]. In fresh tomatoes of the ‘Vulcano’ landrace, the high SAR value was primarily associated with very low TA rather than high TSS content. Indeed, the TSS content of this cultivar is comparable to that of the PA hybrid. Conversely, the high TA in fresh fruits of PA was responsible for the low SAR observed in that cultivar. Citric acid is the most abundant organic acid in tomato fruits; high levels are generally desirable, as they ensure a sufficiently low pH to inhibit bacterial growth and preserve product quality during processing. TA also contributes significantly to the flavour profile of tomato-based products [12]. The low TA content detected in the LS tomato could be attributed to the downregulation of citrate and upregulation of malate observed in the long shelf-life ‘de Penjar’ tomato landrace, which have been associated with reduced respiratory activity [26].

Fresh tomatoes of the LS cultivar also exhibited higher lycopene (Lyco) content, both on a fresh-weight and a dry-weight basis, compared to the two commercial hybrids. It has been reported that carotenoid levels in tomatoes are significantly influenced by the ripening stage and genotype [27]. Previous studies have confirmed the high lycopene content of fruits from this local LS landrace in comparison with commercial hybrids [28]. As previously observed, the elevated Lyco content in LS tomato types is attributed to the thickness of the fruit skin, which accounts for 50–60% of the total lycopene content [1]. Interestingly, the antioxidant activity (AA) of fresh fruits—calculated on both a fresh-weight and a dry-weight basis—showed no variations among the different cultivars. Different antioxidants contributed to similar AA values across the examined tomato cultivars: high levels of ascorbic acid (AscA), in MP and PA, and high levels of Lyco, in LS.

### 4.2. Semi-Dry Product

Semi-dry tomatoes are produced through partial dehydration, a process that significantly reduces their water content while preserving the fruit’s natural juiciness [10]. This partial dehydration enhances both sweetness and aroma, resulting in a semi-dry product that is richer and more concentrated than its fresh counterpart. In the present study, semi-dry tomatoes from the LS landrace exhibited a higher total solids (TS) content compared to non-LS tomatoes. Generally, a high TS content is regarded as a positive attribute in tomatoes, as it imparts greater flavour intensity and a richer taste—qualities highly valued by consumers [29]. In this respect, the semi-dry product obtained from the LS tomato outperformed that derived from non-LS cultivars.

TA of the LS semi-dry tomato was higher than that of the Pachino-type semi-dry tomato when values were expressed on a fresh-weight basis. However, no differences were observed among the cultivars when expressed on a dry-weight basis.

Following dehydration, the LS semi-dry product exhibited a SAR value lower than that of the Pachino-type tomato—renowned for its excellent physicochemical properties—but after one week, these differences were no longer apparent. As previously mentioned, SAR is an important quality parameter that defines tomato sweetness; a high SAR value in the semi-dry LS product, comparable to that of the Pachino-type, can impart high palatability and a pronounced tomato flavour [14].

The reducing sugar (RS) content, expressed on a fresh-weight basis, increased following dehydration due to concentration effects resulting from moisture loss, making the semi-dry product sweeter than its fresh counterpart. When expressed on a dry-weight basis, RS content remained unchanged after dehydration across all cultivars, indicating that the high dehydration temperature did not affect this physicochemical attribute. After one week of storage (T7), RS levels were significantly reduced in the semi-dry product of the Pachino-type but not in that of the LS-type. The lower losses of RS in the LS tomato type can be attributed to its thick cuticle, which limits O_2_/CO_2_ exchange and consequently reduces respiration. Although previous literature supports lower respiration in long-shelf-life tomatoes compared to non-LS cultivars [3], we did not measure respiration rate or gas exchange in the present study.

It is well established that tomatoes play a significant protective role against free radicals, thanks to their high levels of natural antioxidants such as ascorbic acid, polyphenols, and carotenoids [30]. The processing of tomato is generally regarded as the main factor responsible for changes in natural antioxidant levels, with high temperatures causing the greatest degradation [11]. Indeed, oxidative damage can occur during the dehydration process and subsequent storage of tomatoes [31]. In this study, regardless of the cultivars, losses of AscA following dehydration at 80 °C were substantial (−61 and −89%, on a fresh-weight and dry-weight basis, respectively) and more pronounced than those observed for other antioxidants. In fact, when expressed on a fresh-weight basis, total phenol (TP) and flavonoid (Flav) levels increased, while Lyco levels remained unchanged. Fruit AscA degrades markedly at high temperatures [30]. Research on semi-dry cherry tomato has shown that AscA losses are clearly temperature-dependent, increasing with higher dehydration temperatures; the greatest losses were observed in samples dried at 80 °C [11]. However, this high drying temperature was selected based on the study by Muratore et al. [11], who found that carotenoids (lycopene and β-carotene) in semi-dry tomatoes dehydrated at 80 °C for 4 h were better preserved (+30%) compared to those dried at 60 °C for 9 h or 40 °C for 29 h, despite a significant reduction in AscA content. Given that long shelf-life tomatoes are particularly valued for their high lycopene content, this higher temperature was chosen. It is important to note, however, that in the present study, LS semi-dry tomatoes experienced lower losses during storage (−33% AscA in T7) compared to MP and PA samples (AscA losses > 60% in T7), approaching levels found in non-LS semi-dry tomatoes. This suggests a greater potential—compared to commercial hybrids—to maintain antioxidant value over time. Interestingly, TP and Flav contents, like AscA, decreased after dehydration when expressed on a dry-weight basis. This indicates that the increase observed after dehydration on a fresh-weight basis is simply due to a concentration effect; however, the lower content detected on a dry-weight basis in the semi-dry product suggests degradation caused by prolonged exposure to high temperatures (leading to structural collapse [10]) that also affects these antioxidants, albeit to a lesser extent than AscA. In semi-dry LS tomatoes, TP and Flav contents expressed on a dry-weight basis did not differ from those of non-LS tomatoes, indicating a similar antioxidant value in terms of polyphenol content. Flav content in LS semi-dry tomatoes was actually higher when expressed on a fresh-weight basis—a finding attributable to the higher TS content—confirming the high antioxidant value of the product as it stands.

Carotenoids, including lycopene, are sensitive to light and temperature. Indeed, it has been demonstrated that high dehydration temperatures significantly degrade their content. This detrimental effect of heat drying on the lycopene content of semi-dry tomatoes is attributed to the heat-induced isomerisation of the trans-form into the cis-form—the latter being more susceptible to atmospheric oxidation [32]. However, some evidence suggests that lycopene levels remain relatively stable during heat treatment [7,33]. Certain studies have reported a 10% loss of lycopene in tomatoes dried at 110 °C for 4 h, with no loss observed when tomatoes were treated at 80 °C for 8 h [34]. In the present study, when expressed on a fresh-weight basis, Lyco content declined significantly only in LS tomatoes; nevertheless, this value remained higher than that of the non-LS semi-dry tomatoes, even after one week of storage. However, when the data were expressed on a dry-weight basis, a reduction in Lyco content following partial dehydration was observed in all cultivars, indicating a degradative effect of high dehydration temperature on this antioxidant, regardless of the cultivar.

### 4.3. Pearson’s Correlation

The study revealed intriguing correlations between the physicochemical and antioxidant traits examined. When the data were expressed on a fresh-weight basis, a strong positive correlation was observed between TS and RS. A similarly high positive correlation was reported by Anđelini et al. [27] in a collection of eight local Croatian tomato landraces. The strong positive correlation between TP and Flav indicated a close relationship between these bioactive compounds, which were also positively correlated with AA, thereby confirming the findings of Patanè et al. [15] and Cafaro et al. [28]. The absence of any correlation between Lyco and the other variables examined suggested that its accumulation was independent of the other quality parameters.

When data were expressed on a dry-weight basis, weak correlations—both positive and negative—were observed among the quality parameters. The lack of correlation between TSS and the other variables, with the exception of SAR, suggested that this parameter was not associated with most of the antioxidant traits in the analysed dataset. The strong positive correlation between TA and AscA indicated that tomatoes with higher acidity also tended to have higher levels of ascorbic acid. The absence of correlation between RS and SAR and the other traits pointed to a weak association with the biochemical characteristics evaluated. The strong positive correlation found between AscA and AA, TP, and Lyco suggested that vitamin C accumulation is linked to an increase in antioxidant compounds. The moderate positive correlation between Lyco and AA implied that lycopene may contribute to enhancing antioxidant activity, albeit to a lesser extent than TP and AscA.

### 4.4. PCA Analysis

In this study, two principal component analyses (PCA) were conducted: the first based on data expressed on a fresh-weight basis, and the second on data expressed on a dry-weight basis. The first two components (PC1 and PC2) accounted for over 70% of the total variability. The length of the loading vectors indicated the contribution of each variable to explaining sample variability, with longer vectors representing variables that had a more significant influence on the distribution of samples within the PCA space. The analysis revealed that variations caused by dehydration were the primary source of variability, suggesting that partial dehydration had a greater impact on quality parameters than differences among cultivars.

The PCA biplot based on fresh-weight values indicated that Lyco had a minor influence on sample separation, being primarily associated with PC2 rather than PC1. Since PC1 accounted for the majority of the total variance, lycopene content only allowed for a marginal distinction between fresh and semi-dry tomatoes. Lyco was closely associated with the LS fresh tomato samples positioned along PC2. The clustering of fresh tomatoes on the negative side of PC1 highlighted a specific chemical profile characterised by high levels of TA, AscA, and Lyco, but low levels of TP, Flav, and overall physicochemical attributes, compared to semi-dry tomatoes. The opposing positions of fresh samples (on the left side of the score plot) and semi-dry ones (on the right) suggested a progressive decline in TA and AscA following dehydration, making both parameters important markers of freshness.

The placement of semi-dry Pachino-type tomatoes in the upper-right quadrant of the score plot indicated a high physicochemical quality for these samples. Conversely, the position of LS semi-dry tomatoes in the lower-right quadrant of the score plot reflected high quality in terms of both physicochemical attributes (total solids and Brix degree) and antioxidant properties (flavonoid content).

PCA conducted on dry-weight-based data highlighted the high nutritional and antioxidant value of fresh LS samples. Even after dehydration (T0) and subsequent cold storage (T7), LS tomatoes remained distinct from MP and PA samples along PC2, indicating a better retention of certain physicochemical and antioxidant quality attributes following fruit dehydration and during storage.

## 5. Conclusions

The results demonstrated that the semi-drying treatment substantially modified the physicochemical and nutraceutical profile of the tomatoes. The changes observed in the semi-dry product on a fresh-weight basis are attributable to water loss, whereas data based on a dry weight offer a more accurate evaluation of the actual retention or degradation of the bioactive compounds during processing and storage.

This study also suggested that the long shelf-life tomato from the island of Vulcano may be an excellent candidate for producing a semi-dry food product with superior physicochemical and antioxidant qualities. Indeed, compared to the two commercial hybrids examined—including the well-known and highly regarded Pachino-type—the semi-dry product derived from this local landrace showed lower losses during storage in most physicochemical attributes (such as total soluble solids, titratable acidity, total acidity, and reducing sugars) and antioxidant components (including ascorbic acid and flavonoids). The innovation of this product lies primarily in the raw material, the long shelf-life tomato, which could offer significant commercial value due to its ease of preparation and high quality. Semi-dry tomatoes produced from local long shelf-life varieties could represent a novel commercial product, potentially broadening the market offering and increasing the profitability of small and medium-sized enterprises (SMEs) operating in the agri-food sector across Mediterranean regions. However, further sensory, microbiological, technological, and economic studies are necessary to confirm these potential benefits in terms of enhanced commercial value, improved profitability, and extended shelf life.

## Figures and Tables

**Table 1:** Effects of ‘*cultivar*’ on the physicochemical and antioxidant traits of tomato (fresh-weight basis). TS: total solids; TSS: total soluble solids; TA: titratable acidity; RS: reducing sugars; SAR: TSS/TA; AscA: ascorbic acid; TP: total phenols; Flav: flavonoids; Lyco: lycopene; AA: antioxidant activity. Within each row, means (±*SE*) that do not share a letter are significantly different at *p* ≤ 0.05 (Tukey’s test).

Parameter	Cultivar			Average	Significance
	Vulcano	Febo Hy.	Paskualeto Hy.		
TS (g 100 g^−1^)	10.21 ± 0.05 a	9.94 ± 0.27 a	10.38 ± 0.82 a	10.18	ns
TSS (°Brix)	7.10 ± 0.14 a	7.55 ± 0.03 a	7.63 ± 0.27 a	7.43	ns
TA (g citric a. 100 g^−1^)	0.25 ± 0.01 b	0.36 ± 0.01 a	0.33 ± 0.01 a	0.31	***
RS (g 100 g^−1^)	3.06 ± 0.03 b	2.92 ± 0.03 b	3.31 ± 0.09 a	3.10	**
SAR	29.09 ± 1.62 a	21.29 ± 0.43 b	22.75 ± 0.23 b	24.38	**
AscA (mg ascorbic a. 100 g^−1^)	13.13 ± 1.23 b	20.71 ± 0.69 a	21.24 ± 0.61 a	18.36	***
TP (mg GAE 100 g^−1^)	71.34 ± 1.47 b	70.80 ± 2.40 b	87.51 ± 1.80 a	76.57	***
Flav (mg RE 100 g^−1^)	36.84 ± 1.31 a	35.14 ± 0.28 a	27.51 ± 0.71 b	33.16	***
Lyco (mg 100 g^−1^)	8.11 ± 0.21 a	4.09 ± 0.01 b	3.30 ± 0.20 c	5.17	***
AA (μmol TE g^−1^)	1.35 ± 0.02 a	1.33 ± 0.01 a	1.43 ± 0.04 a	1.37	ns

Significant at *p* ≤ 0.01 (**), and 0.001 (***); ns: not significant.

**Table 2:** Effects of ‘*cultivar*’ on the physicochemical and antioxidant traits of tomato (dry-weight basis). TS: total solids; TSS: total soluble solids; TA: titratable acidity; RS: reducing sugars; SAR: TSS/TA; AscA: ascorbic acid; TP: total phenols; Flav: flavonoids; Lyco: lycopene; AA: antioxidant activity. Within each row, means (±*SE*) that do not share a letter are significantly different at *p* ≤ 0.05 (Tukey’s test).

Parameter	Cultivar			Average	Significance
	Vulcano	Febo Hy.	Paskualeto Hy.		
TSS (°Brix)	69.55 ± 1.09 a	76.03 ± 1.81 a	73.97 ± 3.23 a	73.18	ns
TA (g citric a. 100 g^−1^)	2.40 ± 0.10 b	3.57 ± 0.01 a	3.26 ± 0.18 a	3.08	**
RS (g 100 g^−1^)	29.98 ± 0.17 a	29.44 ± 0.49 a	32.13 ± 1.72 a	30.52	ns
SAR	284.88 ± 14.54 a	214.68 ± 10.35 b	221.60 ± 15.43 b	240.39	*
AscA (mg ascorbic a. 100 g^−1^)	128.81 ± 12.66 b	209.03 ± 12.70 a	206.27 ± 10.55 a	181.37	**
TP (mg GAE 100 g^−1^)	698.81 ± 11.12 a	714.50 ± 43.86 a	851.05 ± 50.39 a	754.79	ns
Flav (mg RE 100 g^−1^)	360.80 ± 11.08 a	276.94 ± 6.87 a	343.95 ± 34.33 a	327.23	ns
Lyco (mg 100 g^−1^)	79.42 ± 1.69 a	41.14 ± 0.99 b	32.52 ± 4.55 b	51.03	***
AA (μmol TE g^−1^)	13.18 ± 0.14 a	13.40 ± 0.37 a	13.93 ± 0.69 a	13.50	ns

Significant at *p* ≤ 0.05 (*), 0.01 (**), and 0.001 (***); ns: not significant.

**Table 3:** Main effects of ‘*dehydration*’ (D) and ‘*cultivar*’ (C) on the physicochemical traits of tomato (fresh-weight basis). TS: total solids; TSS: total soluble solids; TA: titratable acidity; RS: reducing sugars; SAR: TSS/TA. For each main effect, means (±*SE*) that do not share a letter are significantly different at *p* ≤ 0.05 (Tukey’s test).

Main Effect		TS (g 100 g^−1^)	TSS (°Brix)	TA (g citric a. 100 g^−1^)	RS (g 100 g^−1^)	SAR
Dehydration (*D*)	Fresh	10.18 ± 0.26 b	7.43 ± 0.12 b	0.31 ± 0.02 a	3.10 ± 0.06 b	24.38 ± 1.29 c
	T0	37.17 ± 1.16 a	28.22 ± 1.33 a	0.13 ± 0.02 c	11.41 ± 0.69 a	284.72 ± 57.89 a
	T7	36.57 ± 1.11 a	28.82 ± 1.27 a	0.16 ± 0.01 b	10.15 ± 0.96 a	193.68 ± 22.85 b
Cultivar (*C*)	Vulcano	30.19 ± 5.05 a	20.75 ± 3.51 b	0.19 ± 0.02 b	9.61 ± 1.73 a	135.22 ± 30.90 b
	Febo Hy.	25.82 ± 3.98 b	19.45 ± 3.01 b	0.24 ± 0.03 a	8.14 ± 1.32 b	101.01 ± 20.79 b
	Paskualeto Hy.	27.91 ± 4.41 b	24.26 ± 4.19 a	0.17 ± 0.04 b	6.90 ± 1.10 b	266.54 ± 70.31 a
*Significance*	*D*	***	***	***	***	***
	*C*	***	***	***	***	***
	*D* × *C*	*	***	***	***	***

Significant at *p* ≤ 0.05 (*) and 0.001 (***).

**Table 4:** ANOVA results for the main effects and interaction on the physicochemical traits of tomato (fresh-weight basis). TS: total solids; TSS: total soluble solids; TA: titratable acidity; RS: reducing sugars; SAR: TSS/TA; *df*: degrees of freedom; *Adj MS*: Adjusted mean square.

Source	*df*	TS	TSS	TA	RS	SAR
		*Adj MS*				
*D*	2	2138.37	1335.39	0.09	180.62	157.094
*C*	2	43.09	55.68	0.01	16.58	68.723
*D* × *C*	4	12.63	27.34	0.01	10.28	26.459
*Error*	18	3.02	1.40	0.00	1.46	1987

*D*: dehydration; *C*: cultivar.

**Table 5:** ANOVA results for the main effects and interaction on the physicochemical traits of tomato (on a dry-weight basis). TSS: total soluble solids; TA: titratable acidity; RS: reducing sugars; SAR: TSS/TA; *df*: degrees of freedom; *Adj MS*: Adjusted mean square.

Source	*df*	TSS	TA	RS	SAR
		*Adj MS*			
*D*	2	69.49	21.58	20.63	628,533
*C*	2	494.47	0.54	62.42	508,241
*D* × *C*	4	218.81	0.35	97.07	230,934
*Error*	18	12.98	0.02	7.28	20,725

*D*: dehydration; *C*: cultivar.

**Table 6:** Main effects of ‘*dehydration*’ (D) and ‘*cultivar*’ (C) on the physicochemical traits of tomato (on a dry-weight basis). TSS: total soluble solids; TA: titratable acidity; RS: reducing sugars; SAR: TSS/TA. For each main effect, means (±*SE*) that do not share a letter are significantly different at *p* ≤ 0.05 (Tukey’s test).

Main Effect		TSS (°Brix)	TA (g 100 g^−1^)	RS (g 100 g^−1^)	SAR
Dehydration (*D*)	Fresh	73.18 ± 1.47 b	3.08 ± 0.18 a	30.52 ± 0.66 a	240.39 ± 13.08 c
	T0	76.78 ± 4.80 ab	0.35 ± 0.06 b	30.59 ± 1.12 a	781.05 ± 169.15 a
	T7	78.65 ± 1.99 a	0.44 ± 0.04 b	27.93 ± 2.69 a	527.24 ± 57.85 b
Cultivar (*C*)	Vulcano	69.16 ± 2.62 c	1.06 ± 0.34 c	31.33 ± 1.17 a	409.37 ± 53.15 b
	Febo Hy.	75.51 ± 1.39 b	1.55 ± 0.50 a	31.06 ± 0.77 a	350.78 ± 39.47 b
	Paskualeto Hy.	83.94 ± 2.92 a	1.25 ± 0.50 b	26.64 ± 2.41 b	788.53 ± 178.67 a
*Significance*	*D*	*	***	ns	***
	*C*	***	***	**	***
	*D* × *C*	***	***	***	***

Significant at *p* ≤ 0.05 (*), 0.01 (**), and 0.001 (***), ns: not significant.

**Table 7:** ANOVA results for the main effects and interaction on the antioxidant traits of tomato (on a fresh-weight basis). AscA: ascorbic acid; TP: total phenols; Flav: flavonoids; Lyco: lycopene; AA: antioxidant activity; *df*: degrees of freedom; *Adj MS*: Adjusted mean square.

Source	*df*	AscA	TP	Flav	Lyco	AA
		*Adj MS*				
*D*	2	566.4	64,151.8	18,950.9	1.38	12.634
*C*	2	61.8	1797.6	2044.2	25.56	0.213
*D* × *C*	4	16.8	223.3	269.9	2.16	0.093
*Error*	18	1.2	63.8	47.0	0.28	0.012

*D*: dehydration; *C*: cultivar.

**Table 8:** Main effects of ‘*dehydration*’ (D) and ‘*cultivar*’ (C) on the antioxidant traits of tomato (on a fresh-weight basis). AscA: ascorbic acid; TP: total phenols; Flav: flavonoids; Lyco: lycopene; AA: antioxidant activity. For each main effect, means (±*SE*) that do not share a letter are significantly different at *p* ≤ 0.05 (Tukey’s test).

Main Effect		AscA (mg ascorbic a. 100 g^−1^)	TP (mg GAE 100 g^−1^)	Flav (mg RE 100 g^−1^)	Lyco (mg 100 g^−1^)	AA (μmol TE g^−1^)
Dehydration (*D*)	Fresh	18.36 ± 1.38 a	76.6 ± 2.91 c	33.2 ± 1.50 b	5.16 ± 0.75 a	1.37 ± 0.02 c
	T0	7.20 ± 1.00 b	233.3 ± 4.85 a	111.9 ± 6.08 a	4.62 ± 0.42 ab	3.74 ± 0.07 a
	T7	3.02 ± 0.21 c	209.3 ± 6.80 b	113.3 ± 6.65 a	4.40 ± 0.40 b	2.57 ± 0.07 b
Cultivar (*C*)	Vulcano	6.61 ± 1.69 c	174.1 ± 26.22 b	102.0 ± 16.44 a	6.57 ± 0.45 a	2.59 ± 0.36 a
	Febo Hy.	11.67 ± 2.48 a	158.4 ± 22.41 c	72.1 ± 11.33 c	4.36 ± 0.10 b	2.39 ± 0.31 b
	Paskualeto Hy.	10.30 ± 2.82 b	186.6 ± 24.91 a	84.3 ± 12.43 b	3.26 ± 0.11 c	2.69 ± 0.36 a
*Significance*	*D*	***	***	***	***	***
	*C*	***	***	***	*	***
	*D* × *C*	***	*	**	***	ns

Significant at *p* ≤ 0.05 (*), 0.01 (**), and 0.001 (***), ns: not significant.

**Table 9:** ANOVA results for the main effects and interaction on the antioxidant traits of tomato (on a dry-weight basis). AscA: ascorbic acid; TP: total phenols; Flav: flavonoids; Lyco: lycopene; AA: antioxidant activity; *df*: degrees of freedom; *Adj MS*: Adjusted mean square.

Source	*df*	AscA	TP	Flav	Lyco	AA
		*Adj MS*				
*D*	2	84,102.5	77,090	1809.7	4521.3	94.38
*C*	2	3187.7	27,637	9628.9	883.6	2.34
*D* × *C*	4	1690.6	2178	820.5	519.4	0.31
*Error*	18	148.8	2508	516.8	9.5	0.30

*D*: dehydration; *C*: cultivar.

**Table 10:** Main effects of ‘*dehydration*’ (D) and ‘*cultivar*’ (C) on the antioxidant traits of tomato (on a dry-weight basis). AscA: ascorbic acid; TP: total phenols; Flav: flavonoids; Lyco: lycopene; AA: antioxidant activity. For each main effect, means (±*SE*) that do not share a letter are significantly different at *p* ≤ 0.05 (Tukey’s test).

Main Effect		AscA (mg ascorbic a. 100 g^−1^)	TP (mg GAE 100 g^−1^)	Flav (mg RE 100 g^−1^)	Lyco (mg 100 g^−1^)	AA (μmol TE g^−1^)
Dehydration (*D*)	Fresh	181.4 ± 14.45 a	754.8 ± 31.07 a	327.2 ± 16.62 a	51.03 ± 7.35 a	13.50 ± 0.26 a
	T0	20.1 ± 3.20 b	630.6 ± 16.23 b	299.5 ± 7.99 b	11.76 ± 0.87 b	10.11 ± 0.29 b
	T7	8.4 ± 0.78 c	573.8 ± 16.94 b	308.2 ± 10.11 b	12.67 ± 1.01 b	7.03 ± 0.10 c
Cultivar (*C*)	Vulcano	48.5 ± 20.42 b	607.0 ± 24.54 b	343.9 ± 6.45 a	36.09 ± 10.85 a	9.70 ± 0.95 b
	Febo Hy.	83.7 ± 31.68 a	637.6 ± 26.48 b	278.5 ± 4.60 c	22.59 ± 4.65 b	10.22 ± 0.91 ab
	Paskualeto Hy.	77.7 ± 32.35 a	714.6 ± 39.82 a	312.6 ± 12.74 b	16.77 ± 4.16 c	10.72 ± 0.99 a
*Significance*	*D*	***	**	***	***	***
	*C*	***	***	ns	***	*
	*D* × *C*	***	ns	ns	***	ns

Significant at *p* ≤ 0.05 (*), 0.01 (**), and 0.001 (***), ns: not significant.

**Table 11:** Eigenvectors (loadings) of the variables (on a fresh-weight and a dry-weight basis) for the first two principal components (PC1 and PC2) obtained from principal component analysis (PCA).

Variable	Fresh-Weight Basis		Dry-Weight Basis	
	PC1	PC2	PC1	PC2
TS	0.351	−0.076	-	-
TSS	0.347	0.085	−0.178	−0.596
TA	−0.334	0.004	0.441	−0.115
RS	0.318	−0.202	0.130	0.371
SAR	0.294	0.341	−0.293	−0.402
AscA	−0.319	0.290	0.439	−0.176
TP	0.355	0.059	0.369	−0.365
Flav	0.342	−0.169	0.187	0.308
Lyco	−0.067	−0.837	0.377	0.096
AA	0.328	0.110	0.407	−0.252

## Data Availability

The original contributions presented in this study are included in the article. Further inquiries can be directed to the corresponding author.
